# Intratumor heterogeneity and cell secretome promote chemotherapy resistance and progression of colorectal cancer

**DOI:** 10.1038/s41419-023-05806-z

**Published:** 2023-05-05

**Authors:** Julia Källberg, Alexandra Harrison, Valerie March, Santa Bērziņa, Ivan Nemazanyy, Oliver Kepp, Guido Kroemer, Sophie Mouillet-Richard, Pierre Laurent-Puig, Valérie Taly, Wenjin Xiao

**Affiliations:** 1 Centre de Recherche des Cordeliers, INSERM, CNRS, Université Paris Cité, Sorbonne Université, USPC, Equipe labellisée Ligue Nationale contre le cancer, Paris, France; 2 Platform for Metabolic Analyses, Structure Fédérative de Recherche Necker, INSERM US24/CNRS UMS 3633, Paris, France; 3 Equipe labellisée par La Ligue contre le cancer, Université Paris Cité, Sorbonne Université, INSERM UMR1138, Centre de Recherche des Cordeliers, Paris, France; 4 Metabolomics and Cell Biology Platforms, Gustave Roussy Cancer Center, Villejuif, France; 5 Institut du Cancer Paris CARPEM, Department of Biology, Hôpital Européen Georges Pompidou, AP-HP, Paris, France; 6 Institut du Cancer Paris CARPEM, Department of Oncology, Hôpital Européen Georges Pompidou, AP-HP, Paris, France

**Keywords:** Tumour heterogeneity, Cancer models

## Abstract

The major underlying cause for the high mortality rate in colorectal cancer (CRC) relies on its drug resistance, to which intratumor heterogeneity (ITH) contributes substantially. CRC tumors have been reported to comprise heterogeneous populations of cancer cells that can be grouped into 4 consensus molecular subtypes (CMS). However, the impact of inter-cellular interaction between these cellular states on the emergence of drug resistance and CRC progression remains elusive. Here, we explored the interaction between cell lines belonging to the CMS1 (HCT116 and LoVo) and the CMS4 (SW620 and MDST8) in a 3D coculture model, mimicking the ITH of CRC. The spatial distribution of each cell population showed that CMS1 cells had a preference to grow in the center of cocultured spheroids, while CMS4 cells localized at the periphery, in line with observations in tumors from CRC patients. Cocultures of CMS1 and CMS4 cells did not alter cell growth, but significantly sustained the survival of both CMS1 and CMS4 cells in response to the front-line chemotherapeutic agent 5-fluorouracil (5-FU). Mechanistically, the secretome of CMS1 cells exhibited a remarkable protective effect for CMS4 cells against 5-FU treatment, while promoting cellular invasion. Secreted metabolites may be responsible for these effects, as demonstrated by the existence of 5-FU induced metabolomic shifts, as well as by the experimental transfer of the metabolome between CMS1 and CMS4 cells. Overall, our results suggest that the interplay between CMS1 and CMS4 cells stimulates CRC progression and reduces the efficacy of chemotherapy.

## Introduction

Colorectal cancer (CRC) is the second most common cancer [1], with metastatic CRC having an extremely low 5-year survival rate of around 15% [2]. Chemotherapeutic drugs such as fluoropyrimidines, especially 5-fluorouracil (5-FU), have been routinely used for the treatment of CRC, alone or in combination with surgery, radiotherapy or targeted treatments [3]. However, chemotherapy resistance represents one of the main obstacles for the effective treatment of CRC [4]. It is therefore important to unravel the molecular mechanisms of drug resistance.

The tumor microenvironment (TME) of CRC is composed of a variety of cell types, including different neoplastic, immune, and stromal cells, as well as blood vessels and elements of the extracellular matrix (ECM), which are in constant interplay [5]. The TME is of cardinal importance for tumor progression, metastasis, and resistance to therapies [6–8]. In addition to interactions between malignant cells and the TME, intratumor heterogeneity (ITH) has also been suggested to contribute to drug resistance of CRC [9–11]. ITH refers to the coexistence of genetically, epigenetically or phenotypically distinct cancer cells within a tumor. Clonal evolution drives the genetic diversification of cancer cells, generating cancer sub-clones [12, 13]. While phenotypic differences between cancer cell types stem from this genomic variation, they can also originate from interactions with the TME, as well as one another [12, 13]. Heterogeneous cancer cells display an inherent functional variability in the proliferative potential that may depend on intercellular communication [10, 14, 15].

Based on transcriptomics data, a recent subtype concordance analysis by the Colorectal Cancer Subtyping Consortium has yielded 4 transcriptionally driven molecular subgroups of tumors,—termed consensus molecular subtypes (CMS) [16]. CMS1 tumors are defined by microsatellite instable/immune features, while CMS2, CMS3, and CMS4 display canonical, metabolic, and mesenchymal phenotypes, respectively. The CMS classification represents a significant advance in understanding CRC inter-tumor heterogeneity, and may serve as a prognostic and predictive factor for the efficacy of therapy against CRC and thus are considered as a path to precision medicine [17]. More recently, research following the derivation of CMS has shown that a tumor can be classified as a mixed CMS, likely reflecting ITH [18–22]. For example, studies on the spatial distribution of CMS in tumors revealed that CMS4 cells are enriched at the tumor ‘invasive front’, while other CMS classes are more frequently found at the core [18, 21]. Intriguingly, our team has observed that more than half of CRC tumors actually correspond to CMS mixtures, highlighting the transcriptional heterogeneity of CRC [22]. Such ITH was associated with dismal prognosis under FOLFOX-based regimen, and this was particularly well documented for tumors composed of CMS1 and CMS4 cells. These findings now raise the question of whether the intratumoral communication between different CMS underlies tumor progression and therapy resistance in CRC. Nevertheless, to the best of our knowledge, such studies have not yet been reported.

The secretome is an emerging mechanism of cellular interplay in tumors, as it contains protumorigenic factors released by different cell types [23]. Compared to their non-malignant counterparts, cancer cells have an aberrant secretome that can influence every stage of the tumorigenic cascade [24]. Importantly, cancer treatments can alter the composition of the cancer cell secretome. Such therapy-induced changes in the secretome can promote the formation of an immunosuppressive TME and tumor relapse [23, 25]. Studies have also shown that the therapy-induced secretome of cancer cells can modulate drug responses in adjacent cells, potentially by stimulating the outgrowth, dissemination, and metastasis of other cancer cell populations [14, 26, 27]. In particular, cells from the core of the tumor can cooperate with those at the invasive front and promote their malignancy by extracellular signals [28]. Thus, research on secretome-dependent mechanisms of cancer cell interplay is essential to expand our current understanding of CRC, from initiation to overcoming therapy resistance.

In this study, we investigated the interaction of human CMS1 and CMS4 cells, and analyzed chemotherapy outcomes. We mimicked the ITH of CRC by coculturing CMS1 and CMS4 cells in a 3D spheroid model. A specific cell distribution pattern was observed in the cocultured spheroids, with CMS1 cells (HCT116 or LoVo) growing at the center, while CMS4 cells (SW620 or MDST8) localizing at the periphery. Although the coculture of CMS1 and CMS4 did not alter the cell growth of either population, CMS1 cells showed a significant drug resistance-promoting effect on their CMS4 counterparts in response to 5-FU, while sustaining their own survival. 5-FU caused CMS1 cells to release factors that stimulated the outgrowth of CMS4 cells. Moreover, such secretome of CMS1 cells supported the invasive capacity of MDST8. Overall, the therapeutic action of 5-FU induced secretome changes of CMS1 cells that promoted 5-FU resistance of tumor spheroids. Of note, we found that secreted metabolites can be responsible for these effects. Altogether, our results provide mechanistic insights into the intercellular communication between CMS1 cells in the tumor spheroid core and edge-located CMS4 cells that may contribute to tumor progression and chemotherapy resistance.

## Results

### 3D tumor spheroid formation of CMS cell lines

HCT116 and LoVo have been classified as CMS1 cells [29], while SW620 and MDST8 have been classified as CMS4 cells [30]. In order to assess the link between CMS and chemotherapy sensitivity, the half-maximal inhibitory concentrations (IC_50_) of 5-FU acting on these cell lines were determined using viability assays. HCT116 cells exhibited the highest sensitivity to 5-FU with IC_50_ = 3.83 ± 0.76 µM for 3 days, whereas SW620 cells were the most resistant with a 32.6-fold higher IC_50_ = 124.68 ± 27.09 µM (Supplementary Table S1), in line with the previous observations that CMS4 cells are relatively resistant against chemotherapy [30, 31].

Tumor spheroids composed of different CMS cell lines were generated using microwell-based cultures with ultralow attachment surfaces. HCT116 cells formed compact spheroids after two days with a diameter of *~*100 μm and grew into *~*450 μm structures on day 4, representing a physiologically relevant size (Supplementary Fig. S1a) [32]. The cells maintained viability for 4 days in culture and exhibited increased 5-FU resistance with IC_50_ = 15.00 ± 3.84 μM (Supplementary Fig. S1b) as compared to 2D monolayer cultures, as previously described [33]. This increased drug resistance is believed to be largely due to the restriction of 5-FU diffusion into 3D structures, as well as due to the molecular concentration gradients in oxygen, pH, nutrients, and cellular metabolites [33, 34]. Although LoVo and MDST8 cells also showed the potential to form spheroids, these structures were rather loose resulting in non-spherical shape (Supplementary Fig. S2). Indeed, the morphology of LoVo spheroids is suggestive of loosely aggregating structures that fail to organize into organoids. MDST8 cells tended to form aggregates of multiple small sub-spheroids that failed to generate compact, fully integrated spheroids. SW620 cells did not adopt a spheroidal conformation at all (Supplementary Fig. S2). Therein, when grown in suspension, distinct CMS cell lines differ in their propensity to generate spheroids.

### Spatial distribution of CMS cells in cocultured 3D spheroids

**Fig. 1:**
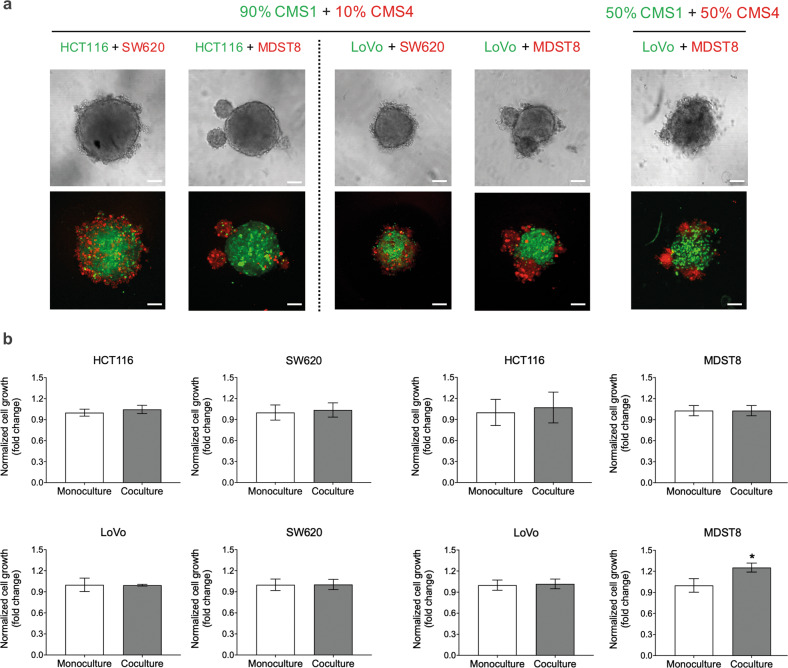
CMS1 and CMS4 cells in cocultured 3D spheroids. **a** Representative live cell confocal fluorescence microscopy images showing spheroid morphology and cell distribution on day 4 post-seeding. HCT116 cells were stained with cell tracker CMFDA (green), SW620 and MDST8 cells were stained with CMRA (red) fluorescent probes, LoVo cells express GFP. Scale bars = 100 μm. **b** Cell growth in cocultured 3D spheroids. Cell growth was measured in cocultured tumor spheroids by means of image analysis quantifying the fluorescence intensity of cell trackers that represent the cell number, and normalized to the monoculture as a control. The bars represent the average of cell growth and the error bars represent the standard deviation (*n* = 3). Statistical significance was calculated using a one-way ANOVA followed by Student’s *t*-test. *P*-values of <0.05 and 0.01 were considered significant (*) and highly significant (**), respectively.

To model the intercellular interactions of CMS populations in vivo, 3D cocultured tumor spheroid models that reflect the ITH of CRC were established. 10% of CMS4 cells, which were either SW620 and MDST8 cells labeled with the CMTRA cell tracker (red), were cocultured with 90% of CMS1 cells such as HCT116 labeled with the CMFDA cell tracker (green) or LoVo expressing green fluorescent protein (GFP). We observed that the CMS1/CMS4 cocultures formed spheroidal structures and that CMS1 cells (HCT116 or LoVo) grew at the center of such spheroids, while CMS4 cells (SW620 or MDST8) preferentially localized in the periphery (Fig. 1a). Similar spheroid morphologies were observed when coculturing CMS1 and CMS4 cells at a 1:1 ratio. Collectively, these data suggest that CMS1 cells present a core-like location while CMS4 cells organize at the edges of mixed spheroids.

### Cell growth and drug resistance in cocultured 3D spheroids

**Fig. 2:**
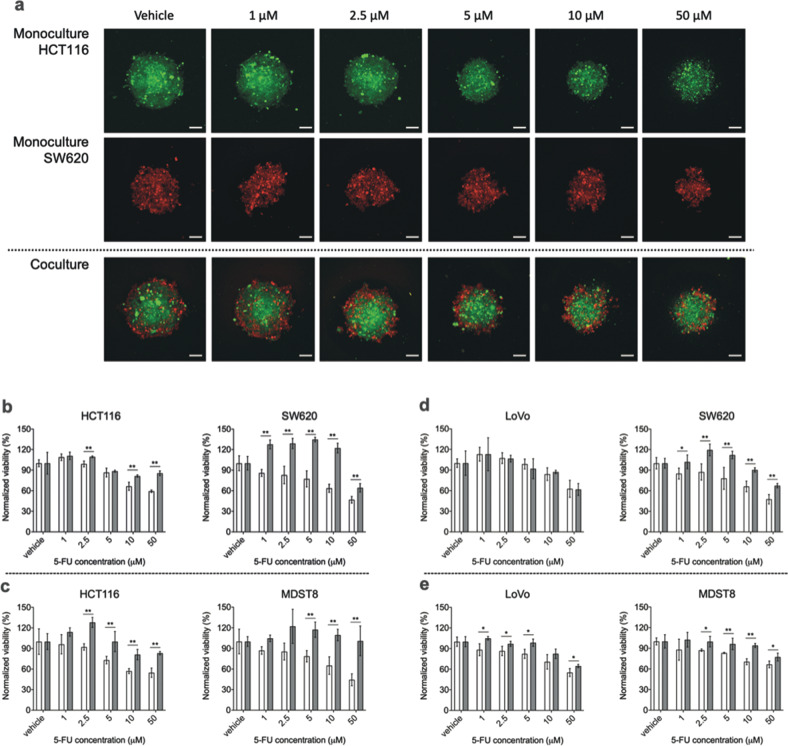
Monocultured and cocultured spheroids of CMS1 and CMS4 cells. **a** Representative live cell confocal fluorescence microscopy images showing the spheroid morphology of HCT116 and SW620 after 3 days of 5-FU treatment. Cells were stained with either cell tracker CMFDA (green) or CMRA (red) fluorescent probes. Scale bars = 100 μm. **b**–**e** 5-FU response of CMS1 and CMS4 cells in spheroids. Monocultured (white bars) and cocultured spheroids (gray bars) of **b** HCT116 and SW620, **c** HCT116 and MDST8, **d** LoVo and SW620 and **e** LoVo and MDST8 were exposed to different concentrations of 5-FU for 3 days. Cell viability was measured by means of image analysis quantifying the fluorescence intensity of cell trackers that represent the cell number, and normalized to the vehicle control. The bars represent the average of viability and the error bars represent the standard deviation (*n* = 3). Statistical significance was calculated using a one-way ANOVA followed by Student’s *t*-test. *P*-values of <0.05 and 0.01 were considered significant (*) and highly significant (**), respectively, when compared to the monoculture.

Next, we explored the effects of CMS interactions on cell growth in cocultured tumor spheroids composed of 10% CMS4 and 90% CMS1 cells. Interestingly, coculture did not appear to exert a strong effect on the cell growth in either population (Fig. 1b). To further assess the effect of coculture on drug resistance, spheroids were treated with 5-FU after their initial formation on day 1 post-seeding. The subsequent growth of each cell population was monitored by fluorescence microscopic imaging. When added to cultures comprising HCT116 cells alone, 10–50 μM of 5-FU decreased the volume of spheroids, accompanied by decreased compactness and shape (Fig. 2a). In contrast, HCT116 cells cocultured with SW620 cells were protected against 5-FU, resulting in an 18.3% and 32.2% increase in HCT116 survival rate with 10 and 50 μM of 5-FU, respectively (Fig. 2b). Moreover, the number of admixed SW620 cells was largely increased, by up to 91% (10 μM of 5-FU), in the coculture compared to that in the monoculture (Fig. 2b). This suggests that CMS1 cells confer 5-FU resistance to CMS4 cells in coculture conditions and that mixed CMS1/CMS4 spheroid possess a collective survival advantage in adverse conditions. Indeed, MDST8 cells were also conferred 5-FU resistance by HCT116 cells. In these mixed spheroids, MDST8 cells showed a 130% increase in survival rate (50 μM of 5-FU) when compared to those in monocultures (Fig. 2c), without being in comprehensive contact with HCT116, but rather forming several small spheroids on their own (Supplementary Fig. S3). The overall survival of HCT116 cells was again supported by MDST8 cells (Fig. 2c). In addition, coculture with HCT116 cells stimulated outgrowth of CMS4 cells against 5-FU treatments, showing a maximum increase of 36% (5 μM of 5-FU) and 22% (2.5 μM of 5-FU) in cell number for SW620 and MDST8, respectively, when compared to the vehicle-treated control.

Finally, LoVo cells stably expressing GFP were cocultured with either SW620 or MDST8 cells. The resulting spheroids were then exposed to different 5-FU concentrations using the same experimental setup as above (Supplementary Figs. S4 and S5). As observed for HCT116 cells, coculture significantly sustained the survival of LoVo and enhanced the resistance of CMS4 cells to 5-FU (Fig. 2d, e). Once again, this effect appeared independent of close contact of one cell population to another (Supplementary Figs. S4 and S5). Collectively, these results suggest that CMS1/CMS4 coculture increases 5-FU resistance.

### The effect of the CMS1 secretome on CMS4 drug resistance

**Fig. 3:**
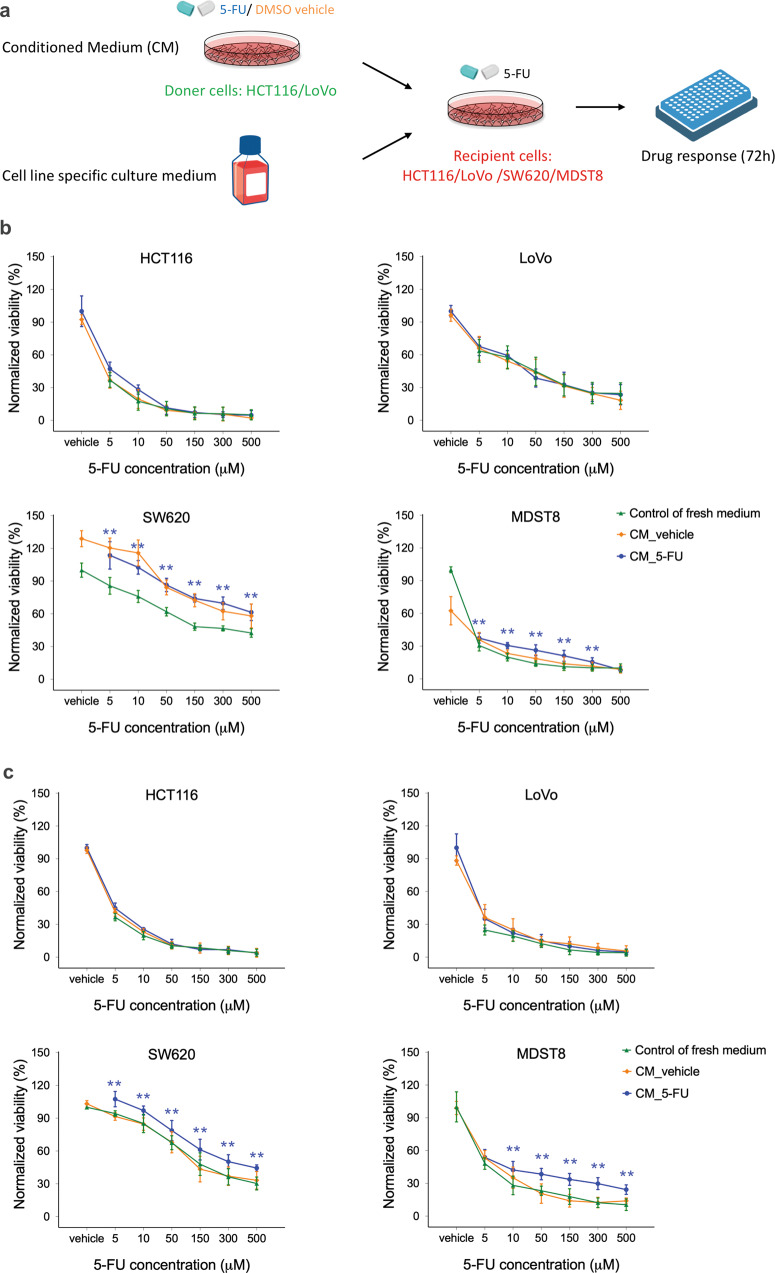
Drug resistance effect of conditioned media (CM) of CMS1 cells. **a** Schematic of recipient cells treated with CM of donor cells. Recipient cells were treated with either CM_vehicle or CM_5-FU of **b** HCT116 or **c** LoVo doner cells, and were exposed to different concentrations of 5-FU for 3 days. Cells treated with media only were taken as a control. Cell viability was measured using MTS assays. The squares, circles, and triangles represent the average viability normalized to the vehicle control and the error bars represent the standard deviation (*n* = 3). Statistical significance was calculated using a one-way ANOVA followed by Student’s *t*-test. *P*-values of <0.05 and 0.01 were considered significant (*) and highly significant (**), respectively, when compared to the control.

We next investigated the potential mechanisms involved in the interplay between CMS1 and CMS4 cells. Recently, Bastola and colleagues reported that the secretome from the glioblastoma core promoted malignancy of cells at the tumor edge [28]. Based on this finding, we examined whether the secretome of 5-FU treated CMS1 cells would influence the drug response of CMS4 cells to 5-FU. Conditioned (CM) were derived from HCT116 cells cultured in the absence (DMSO, CM_vehicle) or presence of 2.5 µM of 5-FU (CM_5-FU) for 3 days. Recipient SW620 cells were then cultured in HCT116 CM and their own culture medium at a 1:1 ratio and exposed to increasing concentrations of 5-FU for 3 days (Fig. 3a). Both CM_vehicle and CM_5-FU dramatically reduced the toxic effect of 500 µM 5-FU on SW620, yielding a threefold increase in viable cells (Fig. 3b). Enhanced resistance to 5-FU used at 30–100 µM was also observed with MDST8 received HCT116 CM. In contrast, the drug response of neither HCT116 nor LoVo was altered by HCT116-derived CM. These data suggest that the HCT116 secretome can promote 5-FU resistance of CMS4 cells specifically.

In an attempt to determine whether the secretome of other CMS1 cells could also induce 5-FU resistance, or whether this phenomenon exclusively applies to HCT116, CM were collected from LoVo cells under the same conditions and added to CMS4 cells. LoVo-derived CM significantly sustained the viability of SW620 and MDST8 cells against 5-FU at concentrations from 10 to 500 μM (Fig. 3c). LoVo CM_vehicle induced minimal or no increase in viability of these cell lines, suggesting that the observed effect is largely the result of specific secretome changes induced by 5-FU. Unlike SW620 and MDST8 cells, HCT116 and LoVo cells were as insensitive to LoVo CM as they were to HCT116 CM (Fig. 3b).

### The effect of the CMS1 secretome on CMS4 migration and invasion

**Fig. 4:**
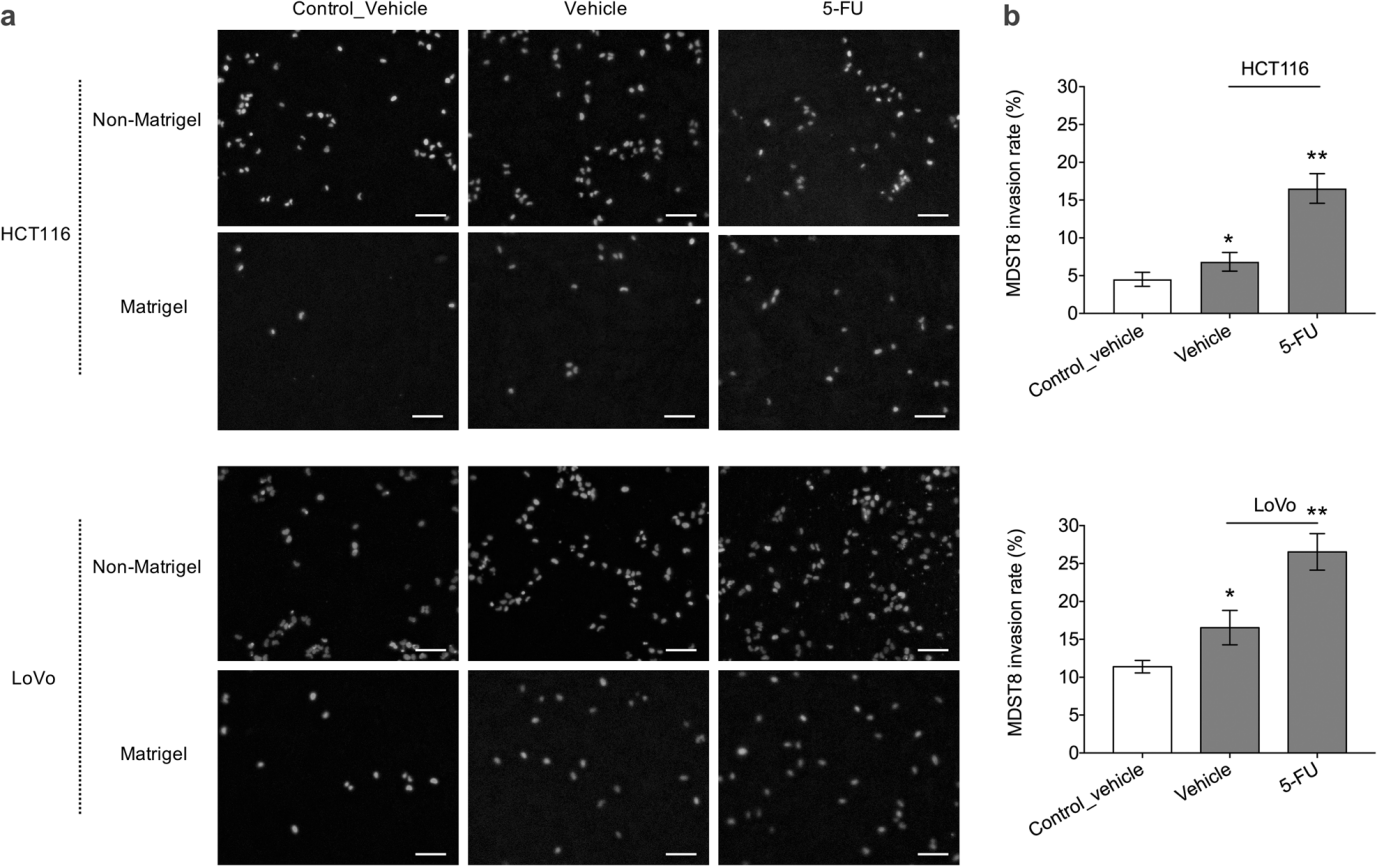
MDST8 cell invasion through transwell membrane. **a** Representative widefield fluorescence microscopy images showing MDST8 migration and invasion through transwell membrane with and without Matrigel coating after 2 day exposure to HCT116 or LoVo in the bottom wells. MDST8 exposed to only media without cells were taken as a control. Cells were treated with either DMSO vehicle or 2.5 μM of 5-FU. Cell nuclei were stained with Hoechst (blue). Scale bars = 100 μm. **b** Invasion rate of MDST8 presented as the percentage of cell invasion through Matrigel-coated transwell membrane relative to the cell migration through the non-Matrigel coated transwell membrane. The bars represent the average and the error bars represent the standard deviation (*n* = 3). Statistical significance was calculated using a one-way ANOVA followed by Student’s *t*-test. *P*-values of <0.05 and 0.01 considered significant (*) and highly significant (**), respectively, when compared to the control.

We next examined the capacity of MDST8 to migrate through the matrix of the basement membrane after exposure to the CMS1 secretome. This was determined using transwell inserts coated with a Matrigel layer onto which MDST8 were cultured. These transwell inserts were then placed on top of HCT116 or LoVo cells. Exposure to soluble signals emanating from HCT116 or LoVo cells modestly increased MDST8 migration through the transwell membrane by 1.15 and 1.23 fold, respectively (Fig. 4a, Fig. S6). DMSO-treated HCT116 and LoVo cells significantly increased MDST8 invasion rate by 1.64 and 1.45 fold, causing 6.82% and 16.54% MDST8 cells to cross the Matrigel barrier, respectively (Fig. 4). The invasion capacity of MDST8 was further promoted by the addition of 5-FU to the system by 16.53% (in response to HCT116 cells) and 26.53% (in response to Lovo cells) (Fig. 4). Therefore, we may surmise that, in response to 5-FU, CMS1 cells secrete factors that promote the invasion capacity of CMS4 cells.

### The effect of metabolites on CMS4 drug resistance

**Fig. 5:**
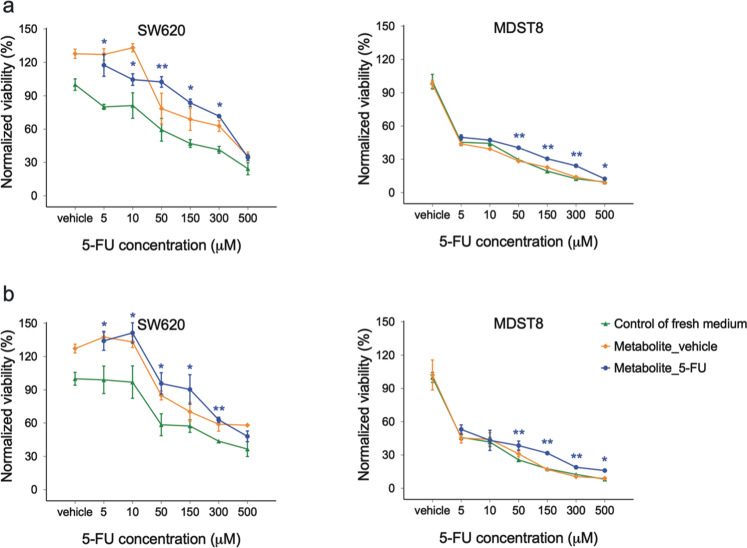
Drug resistance effect of metabolites of CMS1 cells. CMS4 cells were treated with either metabolite_vehicle or metabolite_5-FU of **a** HCT116 or **b** LoVo CMS1 cells, and were exposed to different concentrations of 5-FU for 3 days. Cells treated with media only were taken as a control. Cell viability was measured using MTS assays. The squares, circles, and triangles represent the average viability normalized to the control and the error bars represent the standard deviation (*n* = 3). Statistical significance was calculated using a one-way ANOVA followed by Student’s *t*-test. *P*-values of <0.05 and 0.01 were considered significant (*) and highly significant (**), respectively, when compared to the control.

During tumor progression and metastasis, tumor cells undergo rapid metabolic adaptations and coordinate with their surroundings to maintain biosynthetic and bioenergetic demands while escaping immunosurveillance or therapeutic interventions, which are now recognized as hallmarks of cancer [35]. Thus, we investigated whether metabolites in the CMS1 secretome are responsible for the observed effects. CM were collected from HCT116 or LoVo cultured in the absence (DMSO, CM_vehicle) or presence of 2.5 µM of 5-FU (CM_5-FU) for 3 days. Metabolites of these CM were dialyzed into fresh media (Metabolite_vehicle, Metabolite_5-FU) using dialysis membranes with a cut-off of 3.5 kDa and applied to CMS4 cell lines as previously. Similar to CM_5-FU, Metabolite_5-FU greatly sustained the viability of both SW620 and MDST8 cells against 5-FU at concentrations from 5 to 300 µM (Fig. 5a). The same effect was observed with LoVo metabolites (Fig. 5b). Moreover, we observed the 5-FU resistance-promoting effect with the remaining CM_5-FU after the dialysis of metabolites (Fig. S7). These results suggest that dialyzable metabolites (rather than extracellular vesicles or proteaceous factors) are the key communicators in the CMS1 secretome that can promote 5-FU resistance of CMS4 cells.

### Metabolite analyses of CMS1 conditioned media

To evaluate the metabolic adaptation of CMS1 cells in response to 5-FU, as well as to identify the relevant mediators and pathways involved in the reactive secretome, the CM of CMS1 cells were analyzed by liquid chromatography–tandem mass spectrometry (LC-MS/MS). A total of 146 metabolites involved in a broad range of metabolic pathways were quantified, including amino acids, organic acids, nucleotides, and cofactors (Table S2). The relative steady-state levels of 52 metabolites were significantly altered in the CM_vehicle of HCT116 compared to the control media without cells (Supplementary Fig. S8 and Table 1). Pathway analysis indicated that the levels of 13 metabolites involved in aminoacyl-tRNA biosynthesis (amino acids) were consumed by HCT116 cells, representing the highest pathway significance (Supplementary Table S3). Of note, phenylalanine, tyrosine, and tryptophan biosynthesis, as well as linoleic acid metabolism showed the highest pathway impact of 1.0 among downregulated metabolites. On the other hand, upregulated metabolites in the HCT116 secretome were mainly involved in alanine, aspartate, and glutamate metabolism, including N-acetylaspartate, asparagine, glutamine, fumarate, pyruvate, and alpha-ketoglutarate, representing the highest pathway significance (Supplementary Table S4). Moreover, D-glutamine and D-glutamate metabolism and vitamin B6 metabolism showed the highest pathway impact of 0.50 and 0.49, respectively.

**Table 1:** Fold change of metabolite levels in DMSO vehicle-treated conditioned media (CM_Vehicle) of HCT116 compared to control media without cells (Control).

Metabolite	log2 (fold change, downregulated)	Metabolite	log2 (fold change, upregulated)
Hypoxanthine	−6.49	S-Adenosyl-L-Homocysteine	7.03
Niacin/ Nicotinate	−5.64	Glycerol 3-phosphate	5.94
Linoleic acid	−4.79	Alpha-Ketoglutarate	3.95
Cytidine	−4.69	Orotic acid	3.80
Glutamine	−4.45	Pyruvate	3.25
Adenine	−2.95	Lactate	2.91
Serine	−2.28	N-acetylaspartate	2.85
Taurine	−1.90	Serotonin	1.59
Oleic acid	−1.74	Pyridoxal	1.26
Hexanoylcarnitine	−1.51	Acetyllysine	0.94
Palmitoleic acid	−1.44	Decanoic acid	0.70
Tryptophan	−1.39	Nicotinamide	0.65
Creatine	−1.39	Malate	0.61
Cystine	−1.14	Asparagine	0.57
Leucine	−1.01	Fumarate	0.45
Lysine	−0.99	Urate	0.40
Methionine	−0.94	Glutamate	0.40
Acetylglutamine	−0.92	Glycine	0.34
Ornithine	−0.88	L-Kynurenine	0.24
Valine	−0.84	Fructose	0.20
Threonine	−0.80	Creatinine	0.19
Glucose	−0.69		
Tyrosine	−0.66		
L-Alanine	−0.50		
L-Sarcosine	−0.50		
Histidine	−0.50		
Phenylalanine	−0.49		
Cysteine sulfinic acid	−0.47		
IsoLeucine	−0.46		
Carnitine	−0.45		
Aspartate	−0.36		

The levels of 51 metabolites were significantly altered in the CM_vehicle of LoVo compared to the control media without cells (Supplementary Fig. S9 and Table 2). Similar to HCT116, significantly downregulated metabolites were involved in the aminoacyl-tRNA biosynthesis, representing the highest pathway significance (Table 2, Supplementary Table S5). Once again, phenylalanine, tyrosine, and tryptophan biosynthesis together with linoleic acid metabolism showed the highest pathway impact. Unlike HCT116 cells, upregulated metabolites were mainly involved in citrate cycle (TCA cycle), representing the highest pathway significance (Supplementary Table S6). Riboflavin metabolism, D-glutamine and D-glutamate metabolism, and vitamin B6 metabolism showed the highest pathway impact of 0.50, 0.50, and 0.49, respectively. Overall, the metabolite profile of LoVo CM_vehicle largely overlaps with that of HCT116 CM_vehicle (Supplementary Table S7).

**Table 2:** Fold change of metabolite levels in DMSO vehicle-treated conditioned media (CM_Vehicle) of LoVo compared to control media without cells (Control).

Metabolite	log2 (fold change, downregulated)	Metabolite	log2 (fold change, upregulated)
Docosahexaenoic acid	−6.75	Acetylcysteine	9.11
Niacin/ Nicotinate	−6.73	Orotic acid	6.29
Hypoxanthine	−6.48	Alpha-Ketoglutarate	4.10
Cytidine	−5.38	N-Acetylaspartate	3.54
Glutamine	−4.78	Glycerol 3-phosphate	3.45
Linoleic acid	−2.89	Lactate	2.97
Serine	−2.53	Pyruvate	2.86
Taurine	−2.09	Cysteine	2.23
Tryptophan	−2.05	Butyric acid	2.06
Palmitoleic acid	−1.91	Serotonin	1.99
Creatine	−1.71	Pyridoxal	1.93
Oleic acid	−1.43	Acetyl-lysine	1.67
L-Alanine	−1.32	Cis-aconitate	1.35
L-Sarcosine	−1.32	Nicotinamide	1.02
Methionine	−1.29	Butyryl-carnitine	0.92
Threonine	−0.98	Malate	0.72
Lysine	−0.85	Fumarate	0.53
Tyrosine	−0.85	L-Kynurenine	0.40
Glucose	−0.83	Citrulline	0.33
Cysteinesulfinic acid	−0.76	Riboflavin	0.29
Carnitine	−0.75	Acetylcarnitine	0.28
IsoLeucine	−0.73	Creatinine	0.24
Aspartate	−0.72	Glycine	0.22
Leucine	−0.68	Pantothenate	0.22
Phenylalanine	−0.67	Glutamate	0.22
Histidine	−0.65		

We next examined the influence of 5-FU on the metabolite composition of CMS1 secretome. A total of 37 soluble metabolites exhibited differential patterns in the secretome induced by 5-FU compared to vehicle for both HCT116 and LoVo (Supplementary Figs. S10 and S11, Table S8 and S9). Among these metabolites, we observed a significant overlap of 22 (19 upregulated and 3 downregulated) compounds between HCT116 and LoVo (Table 3). Pathway analysis on these upregulated metabolites revealed that 5-FU treatments impacted several metabolic pathways, including aminoacyl-tRNA biosynthesis, which showed the highest pathway significance. Phenylalanine, tyrosine, and tryptophan biosynthesis and linoleic acid metabolism had the highest pathway impact of 1.0 (Supplementary Table S10). These data suggest that such differentially regulated factors in the CMS1 secretome, induced by 5-FU, could stimulate drug resistance, outgrowth, and invasion capacity of CMS4 cells.

**Table 3:** Overlap of fold change of metabolite levels in 5-FU treated conditioned media (CM_5-FU) of HCT116 and LoVo compared to DMSO vehicle-treated conditioned media (CM_Vehicle).

Metabolite	log2 (fold change, downregulated)		Metabolite	log2 (fold change, upregulated)	
	HCT116	LoVo		HCT116	LoVo
Lactate	−1.01	−0.93	Glutamine	4.02	4.03
Acetyllysine	−0.53	−0.79	Niacin/ Nicotinate	2.96	1.85
Fructose	−0.20	−0.24	Linoleic acid	2.81	1.78
			Hypoxanthine	2.24	1.65
			Palmitoleic acid	1.74	1.65
			Serine	1.66	1.51
			Oleic acid	1.55	1.49
			Tryptophan	1.18	1.26
			Lysine	0.88	1.26
			Creatine	0.84	1.09
			Pyruvate	0.79	1.05
			Methionine	0.71	0.93
			Threonine	0.56	0.78
			Tyrosine	0.50	0.77
			L-Alanine	0.44	0.76
			L-Sarcosine	0.44	0.70
			Cytidine	0.42	0.55
			Phenylalanine	0.37	0.50
			IsoLeucine	0.33	0.40

### The effect kynurenine pathway (Kyn) metabolites on CMS4 drug resistance

Following the metabolite analyses of CMS1 CM, we further tested the functional significance of the corresponding metabolites. We observed that phenylalanine, tyrosine and tryptophan biosynthesis and linoleic acid metabolism were highly upregulated in the CMS1 secretome in response to 5-FU. Tryptophan metabolism occurs mainly via the Kyn pathway, which has been shown to promote colorectal cancer progression, especially by enhancing cellular proliferation and resistance to apoptosis [36, 37]. Therefore, we evaluated the effect of 5 metabolites involved in the Kyn pathway on CMS4 drug resistance [38]. Nicotinamide, kynurenine, and quinolinic acid sustained the viability of SW620 cells against 150 µM of 5-FU (Fig. S12). A similar drug resistance-promoting effect was also observed with nicotinamide and kynurenic acid on MDST8 cells responding to 50 and 150 µM of 5-FU (Fig. S13). These results suggest that the metabolites from Kyn pathway in the CMS1 secretome might contribute to promote 5-FU resistance of CMS4 cells.

## Discussion

ITH facilitates therapeutic resistance in CRC. In this study, we demonstrate an impact of the interplay between CMS1 and CMS4 cell lines on CRC drug resistance and progression. Investigation of the intercellular communication between these two cancer cell populations uncovers that the secretome, specifically the metabolites, from CMS1 cells promotes CMS4 chemotherapy resistance, outgrowth, and invasion. Our findings provide new evidence that the inter-clonal communication occurs between CRC cancer cells and such interplay can confer tumor aggressiveness. This work also highlights distinct secretive factors involved in the heterogenous clonal cooperation, which could represent potential targets for preventing tumor progression.

Two mechanisms could underlie the observed increase in the drug resistance of CMS cells: cell interplay and/or direct consequences of 5-FU exposure. The latter may involve the 5-FU-driven selection and proliferation of drug-resistant subclones or a ‘shielding’ effect caused by the preferential 5-FU targeting of CMS1 cells over CMS4 cells [39, 40]. Such an effect is more likely for the highly 5-FU-sensitive HCT116 cells rather than LoVo cells. Given the high seeding cell density of HCT116 cells in cocultures, their 5-FU-induced demise could consequently result in an increase in available space and nutrients, prompting the growth of CMS4 cells. However, we did not observe any enhanced drug resistance in monocultures of CMS4 cells without HCT116. Therefore, cell interplay is more likely the cause of the observed increase in resistance.

Cell communication may involve direct physical interactions and/or secreted signaling among different cell types. CRC cells have been reported to secrete certain factors into the extracellular space, allowing their communication with the microenvironment [41]. Here, we proved that secreted signals can also be shuttled from one cancer cell type to another, thus affecting therapy resistance, outgrowth, and invasion. This finding indicates that direct cell-to-cell contact may not be obligatory for such effects [28, 42–44]. Secreted signals may also account for previously reported mechanisms, including the paracrine modulation of cellular resistance to chemotherapy-induced cell death [23]. Chemoresistance can also be conferred between tumor cells through the secretome by upregulating the expression of drug efflux pumps and antiapoptotic proteins [23]. Indeed, we found that the composition of the secretome was significantly altered after administration of 5-FU. Components of such therapy-induced secretome have been reported to allow cancer cells to interact with various non-malignant cell types surrounding them, like immune cells [45], and to promote epithelial-to-mesenchymal transition in pre-malignant and malignant cells [46].

The secretome is a complex network of secreted signals, the major components of which include extracellular vesicles (EVs) and soluble factors, such as cytokines, growth factors, enzymes, and metabolites [23]. By interacting with surrounding cells, cancer cell-derived EVs can promote CRC progression, drug resistance, escape of immune-surveillance, angiogenesis, invasion and metastasis [47–49]. Soluble mediators secreted from cancer cells, such as cytokines, growth factors and enzymes, have also been reported to strongly correlate with tumor recurrence and compromised therapeutic efficacy in various cancers [23]. In the context of raising knowledge on cancer-cell-intrinsic metabolic remodeling, recent studies have begun to explore metabolic communications between tumor cells and TMEs and their effect on therapeutic interventions [50–53]. The release of metabolites, such as saccharides, amino acids, lipids, and nucleosides, can induce specific pathways in neighboring non-malignant cells, thereby modulating TMEs. For example, the secreted metabolites of fibrosarcoma cells have been reported to induce vascular tube formation of endothelial cells, resulting in pro-metastatic angiogenesis [54]. However, such metabolic communications between heterogenous populations of cancer cells have remained underexplored. Therefore, as a preliminary study of the cell communication between CMS of CRC, we examined a limited set of soluble metabolites, selected from a broad range of major pathways, followed by a specific focus on the Kyn pathway. In the future, this investigation should be extended to in-depth examination of the metabolomics that may help to uncover new strategies for alleviating therapy resistance in CRC.

## Materials and methods

### Cell culture

Human CRC cell lines were obtained from ATCC or ECACC. All cell lines were maintained in culture flasks (Corning, USA) in a humid 5% CO_2_ incubator at 37 °C. HCT116 (ATCC®CCL-247), LoVo (ATCC®CCL-229), MDST8 (ECACC99011801), and SW620 (ATCC®CCL-227) cells were respectively maintained in McCoy’s 5 A, F-12K, DMEM, and Leibovitz’s L-15(Gibco, USA). All culture media were supplemented with 10% (v/v) inactivated FBS (Gibco, France) and 1% (v/v) penicillin-streptomycin (Gibco, France), and were changed every 3 days.

### IC50 determination

IC50 values of 5-FU (Sigma, US) were determined for all cell lines. Cells were seeded into 96-well plates and incubated overnight to allow attachment, followed by 5-FU treatments in various concentrations of 0, 0.5, 1.0, 1.5, 2.5, 5,0, 10, 20, 30, 50, 75, 100, and 150 µM for 3 days. Cell viability was assayed using 3-(4,5-dimethylthiazol-2-yl)-5-(3carboxymethoxyphenyl)-2-(4-sulfophenyl)-2H-tetrazolium (MTS) assay (Promega, USA), before being washed once with culture media to remove any drug residue. After a 4 h incubation, the absorbance at 570 nm (600 nm as a reference) was measured using a plate reader (Tecan, Switzerland). IC50 values were calculated by using Prism 9.

### 3D spheroids generation and 5-FU treatments

One thousand five hundred cells were seeded into ultralow attachment U-shaped 96-well plates (Corning, USA). Cell seeding was followed by an overnight incubation to allow tumor spheroid formation. Spheroids were treated with 0, 1, 2.5, 5, 10, and 50 µM of 5-FU for 3 days. Cell viability was assayed using CellTiter 96® AQueous One Solution Cell Proliferation Assay (MTS; Promega, USA), before being washed once with culture media to remove any drug residue. After a 4 h incubation, the absorbance at 490 nm was measured using a plate reader (Tecan, Switzerland).

### Live-cell confocal fluorescence imaging and image analyses

A Zeiss LSM710 confocal laser scanning microscope was used for live-cell imaging. Cells were stained with 0.5 µM of either cell tracker CMFDA (green) or CMRA (red) fluorescent probes (Life Technologies, USA), following the manufacturer’s instructions. LoVo cells were transduced with LentiBrite Lentiviral Biosensor (Sigma, USA) to express H2B-GFP. The excitation filters used were 450–490 nm for cells labeled with CMFDA or expressing GFP and 515–560 nm for CMRA. Images were captured using LSM Zen Black software (Zeiss, Germany) and processed by Fiji software. Area and mean pixel intensity measurements of images were taken on each fluorescence channel with B&W threshold on a dark background. These parameters were used to calculate integrated density indicating cell area.

### Cell migration and invasion assay

BioCoat GFR Matrigel invasion inserts (Corning, USA) were rehydrated following the manufacturer’s instructions. 1 × 10^4^ cells in culture medium containing 0.1% BSA were seeded into 24-well plates. The rehydrated GFR Matrigel invasion inserts were next transferred to the wells and 1 × 10^4^ cells were seeded to the inside of these inserts. BioCoat control inserts without GFR Matrigel coating (Corning, USA) were taken as controls. Cells were incubated in a humid 5% CO_2_ incubator at 37 °C for 24 h to allow migration. After incubation, the non-invading cells were removed from the upper surface of the insert membrane following the manufacturer’s instructions and then the nuclei of invading cells were stained with Hoechst (Life Technologies, USA). The cells were imaged by using a Zeiss Axio Observer Z1 widefield microscope (Zeiss, Germany) and processed by Fiji software. Cell counting was performed on obtained images and the number of cells was calculated as a mean average of 9 images per condition. The percent of invading cells were defined as Eq. 1.

1 $${{{\mathrm{\% }}}}\,Invasion = \frac{{Number\,of\,cells\,invading\,through\,GFR\,Matrigel\,insert\,membrane}}{{Number\,of\,cells\,migrating\,through\,control\,insert\,membrane}} \times 100$$

### Conditioned media (CM) collection

HCT116 or LoVo cells were seeded into T-75 cm^2^ flasks and incubated overnight to allow attachment. Media was then replaced with 2.5 µM of 5-FU or DMSO vehicle as a control. After a 3-day incubation, the conditioned media (CM) were collected and centrifuged at 200 rcf for 5 min to remove any cells. The supernatant was then filtered using a syringe and 0.22 µm filters (Sartorius, Germany), flash-frozen and stored at −80 °C.

### Conditioned media (CM) treatment

1500, 10,000, 2000, and 2000 cells of HCT116, LoVo, MDST8, and SW620, respectively, were seeded into 96-well plates and left to incubate overnight. The cell treatment was carried out by diluting 5-FU solutions in a 1:1 mixture of cell line specific culture media and HCT116 or LoVo CM. Final 5-FU concentrations were 5, 10, 50, 150, 300, and 500 µM. After 3 days of treatment, cell viability was assayed using MTS (Promega, USA).

### Metabolite dialysis and treatment

CM of HCT116 or LoVo cells were collected as previously described and centrifuged at 200 rcf for 5 min to remove any cells. Metabolites of the CM were dialyzed into fresh media using 3.5 K MWCO cellulose dialysis tubing (ThermoFisher Scientific, USA) at 4 °C. 2000 cells of MDST8 and SW620, respectively, were seeded into 96-well plates and left to incubate overnight. The cell treatment was carried out by diluting 5-FU solutions in a 1:1 mixture of cell line-specific culture media and HCT116 or LoVo metabolite solution. Final 5-FU concentrations were 5, 10, 50, 150, 300, and 500 µM. After 3 days of treatment, cell viability was assayed using MTS (Promega, USA).

### Targeted metabolites analyses by liquid chromatography–tandem mass spectrometry (LC-MS/MS)

CM metabolites were extracted as previously described [55]. The extraction solution was composed of 50% methanol, 30% ACN, and 20% water. CM samples were diluted 30-fold by adding an extraction solution. Samples were vortexed for 5 min at 4 °C and then centrifuged at 16,000 rcf for 15 min at 4 °C. The supernatants were collected and stored at −80 °C until analyses. LC/MS analyses were conducted on a QExactive Plus Orbitrap mass spectrometer equipped with an Ion Max source and a HESI II probe and coupled to a Dionex UltiMate 3000 UPLC system (ThermoFisher Scientific, USA). External mass calibration was performed using the standard calibration mixture every 7 days as recommended by the manufacturer. 5 μL of each sample was injected onto Zic‐pHilic (150 mm × 2.1 mm i.d. 5 μm) with the guard column (20 mm × 2.1 mm i.d. 5 μm) (Millipore, USA) for the liquid chromatography separation. Buffer A was 20 mM ammonium carbonate, 0.1% ammonium hydroxide (pH 9.2); buffer B was acetonitrile. The chromatographic gradient was run at a flow rate of 0.200 μL/min as follows: 0–20 min; linear gradient from 80 to 20% B; 20–20.5 min; linear gradient from 20% to 80% B; 20.5–28 min: hold at 80% B [55]. The mass spectrometer was operated in full scan, polarity switching mode with the spray voltage set to 2.5 kV, the heated capillary held at 320 °C. The sheath gas flow was set to 20 units, the auxiliary gas flow was set to 5 units, and the sweep gas flow was set to 0 unit. The metabolites were detected across a mass range of 75-1 000 m/z at a resolution of 35,000 (at 200 *m/z*) with the AGC target at 106, and the maximum injection time at 250 ms. Lock masses were used to ensure mass accuracy below 5 ppm. Data were acquired with Thermo Xcalibur software (ThermoFisher Scientific, USA). The peak areas of metabolites were determined using Thermo TraceFinder software (ThermoFisher Scientific, USA), identified by the exact mass of each singly charged ion and by known retention time on the HPLC column. Metabolomic data analyses were performed using Metaboanalyst 5.0 software [56].

### Kynurenine (Kyn) pathway metabolite treatment

2000 of MDST8 and SW620 cells were seeded into 96-well plates and left to incubate overnight. The cells were treated with different concentrations of 5-FU in combination with tryptophan, nicotinamide, kynurenine, kynurenic acid, and quinolinic acid (Sigma, USA). The final 5-FU concentrations were 10, 50 and 150 µM, while final metabolite concentrations were 100 µM and 1 mM. After 3 days of treatment, cell viability was assayed using MTS (Promega, USA).

### Statistical analysis

Unless otherwise mentioned, data are reported as means ± standard deviation of triplicate determinations, and experiments were repeated at least three times yielding similar results. Statistical significance was determined by a one-way ANOVA followed by two-tailed equal variance Student’s *t*-test. *P*-values <0.05 and 0.01 were considered significant (*) and highly significant (**), respectively.

## Supplementary information

Supplemental tables and figures

Supplemental table S2

Reproducibility checklist

## Data Availability

The datasets generated during and/or analyzed during the current study are available from the corresponding author on reasonable request.
