# Supplementary material for: Intratumor heterogeneity and cell secretome promote chemotherapy resistance and progression of colorectal cancer
Source: Cell Death Dis. 2023 May 5;14(5):306. doi: 10.1038/s41419-023-05806-z (PMC10160076; doi:10.1038/s41419-023-05806-z)
Supplement: Supplementary file 1 — Supplemental tables and figures [file 41419_2023_5806_MOESM1_ESM.pdf]

Table S1 IC_{50} of colorectal cancer cell lines to 5-FU

Colorectal cancer (CRC) cell lines were treated with different concentrations of 5-FU and cell viability was measured using MTS assays. The average and the standard deviation were calculated using cells from 3 different passages (n=3). Statistical significance was calculated using a two-tailed Student’s t-test. P-values of less than 0.05 and 0.01 were considered significant (*) and highly significant (**), respectively.

Cell line	IC_{50} (μM) of 72 h
HCT116	3.83 ± 0.76
LoVo	18.85 ± 3.65
MDST8	8.53 ± 1.60
SW620	124.68 ± 27.09

Table S3. Pathway analysis on down-regulated metabolites in DMSO vehicle treated conditioned media (CM_Vehicle) of HCT116 compared to control media without cells (Control).

The Total is the total number of compounds in the pathway; the Hits is the actually matched number from the user uploaded data; the Raw p is the original p value calculated from the enrichment analysis; the Holm p is the p value adjusted by Holm-Bonferroni method; the FDR p is the p value adjusted using False Discovery Rate; the Impact is the pathway impact value calculated from pathway topology analysis.

	Total	Expected	Hits	Raw p	-log10(p)	Holm adjust	FDR	Impact
Aminoacyl-tRNA biosynthesis	48	0.90	13	2.04E-13	1.27E+01	1.71E-11	1.71E-11	0.00
Valine, leucine and isoleucine biosynthesis	8	0.15	4	6.59E-06	5.18E+00	5.47E-04	2.77E-04	0.00
Arginine biosynthesis	14	0.26	3	1.87E-03	2.73E+00	1.53E-01	4.16E-02	0.06
Phenylalanine, tyrosine and tryptophan biosynthesis	4	0.07	2	1.98E-03	2.70E+00	1.61E-01	4.16E-02	1.00
Taurine and hypotaurine metabolism	8	0.15	2	8.83E-03	2.05E+00	7.06E-01	1.48E-01	0.71
Phenylalanine metabolism	10	0.19	2	1.39E-02	1.86E+00	1.00E+00	1.69E-01	0.36
Alanine, aspartate and glutamate metabolism	28	0.52	3	1.41E-02	1.85E+00	1.00E+00	1.69E-01	0.34
Glycine, serine and threonine metabolism	33	0.62	3	2.20E-02	1.66E+00	1.00E+00	2.06E-01	0.09
Cysteine and methionine metabolism	33	0.62	3	2.20E-02	1.66E+00	1.00E+00	2.06E-01	0.13
Nicotinate and nicotinamide metabolism	15	0.28	2	3.05E-02	1.52E+00	1.00E+00	2.56E-01	0.00
Histidine metabolism	16	0.30	2	3.45E-02	1.46E+00	1.00E+00	2.56E-01	0.22
Valine, leucine and isoleucine degradation	40	0.75	3	3.66E-02	1.44E+00	1.00E+00	2.56E-01	0.00
Pantothenate and CoA biosynthesis	19	0.36	2	4.75E-02	1.32E+00	1.00E+00	3.07E-01	0.00
beta-Alanine metabolism	21	0.39	2	5.70E-02	1.24E+00	1.00E+00	3.42E-01	0.00
Linoleic acid metabolism	5	0.09	1	9.02E-02	1.04E+00	1.00E+00	5.05E-01	1.00
D-Glutamine and D-glutamate metabolism	6	0.11	1	1.07E-01	9.69E-01	1.00E+00	5.30E-01	0.00
Nitrogen metabolism	6	0.11	1	1.07E-01	9.69E-01	1.00E+00	5.30E-01	0.00
Purine metabolism	65	1.22	3	1.19E-01	9.26E-01	1.00E+00	5.53E-01	0.02
Biosynthesis of unsaturated fatty acids	36	0.67	2	1.44E-01	8.41E-01	1.00E+00	6.26E-01	0.00
Ubiquinone and other terpenoid-quinone biosynthesis	9	0.17	1	1.57E-01	8.05E-01	1.00E+00	6.26E-01	0.00
Arginine and proline metabolism	38	0.71	2	1.57E-01	8.03E-01	1.00E+00	6.26E-01	0.12
Pyrimidine metabolism	39	0.73	2	1.64E-01	7.85E-01	1.00E+00	6.26E-01	0.01
Biotin metabolism	10	0.19	1	1.73E-01	7.63E-01	1.00E+00	6.30E-01	0.00
Selenocompound metabolism	20	0.37	1	3.16E-01	5.00E-01	1.00E+00	1.00E+00	0.00
Lysine degradation	25	0.47	1	3.79E-01	4.22E-01	1.00E+00	1.00E+00	0.00
Glycolysis / Gluconeogenesis	26	0.49	1	3.90E-01	4.08E-01	1.00E+00	1.00E+00	0.00
Glutathione metabolism	28	0.52	1	4.13E-01	3.84E-01	1.00E+00	1.00E+00	0.00
Glyoxylate and dicarboxylate metabolism	32	0.60	1	4.57E-01	3.40E-01	1.00E+00	1.00E+00	0.00
Tryptophan metabolism	41	0.77	1	5.44E-01	2.65E-01	1.00E+00	1.00E+00	0.14
Tyrosine metabolism	42	0.79	1	5.52E-01	2.58E-01	1.00E+00	1.00E+00	0.14
Primary bile acid biosynthesis	46	0.86	1	5.86E-01	2.32E-01	1.00E+00	1.00E+00	0.01

Table S4. Pathway analysis on up-regulated metabolites in DMSO vehicle treated conditioned media (CM_Vehicle) of HCT116 compared to control media without cells (Control).

The Total is the total number of compounds in the pathway; the Hits is the actually matched number from the user uploaded data; the Raw p is the original p value calculated from the enrichment analysis; the Holm p is the p value adjusted by Holm-Bonferroni method; the FDR p is the p value adjusted using False Discovery Rate; the Impact is the pathway impact value calculated from pathway topology analysis.

	Total	Expected	Hits	Raw p	-log10(p)	Holm adjust	FDR	Impact
Alanine, aspartate and glutamate metabolism	28	0.38	6	8.91E-07	6.05E+00	7.48E-05	7.48E-05	0.33
Citrate cycle (TCA cycle)	20	0.27	4	1.05E-04	3.98E+00	8.72E-03	4.36E-03	0.18
Pyruvate metabolism	22	0.30	4	1.56E-04	3.81E+00	1.28E-02	4.36E-03	0.24
Glyoxylate and dicarboxylate metabolism	32	0.43	4	7.01E-04	3.15E+00	5.68E-02	1.19E-02	0.11
Arginine biosynthesis	14	0.19	3	7.10E-04	3.15E+00	5.68E-02	1.19E-02	0.12
D-Glutamine and D-glutamate metabolism	6	0.08	2	2.54E-03	2.60E+00	2.01E-01	3.55E-02	0.50
Butanoate metabolism	15	0.20	2	1.65E-02	1.78E+00	1.00E+00	1.98E-01	0.00
Aminoacyl-tRNA biosynthesis	48	0.65	3	2.51E-02	1.60E+00	1.00E+00	2.63E-01	0.00
Glycolysis / Gluconeogenesis	26	0.35	2	4.67E-02	1.33E+00	1.00E+00	4.36E-01	0.10
Glutathione metabolism	28	0.38	2	5.35E-02	1.27E+00	1.00E+00	4.49E-01	0.11
Porphyrin and chlorophyll metabolism	30	0.41	2	6.06E-02	1.22E+00	1.00E+00	4.63E-01	0.00
Cysteine and methionine metabolism	33	0.45	2	7.18E-02	1.14E+00	1.00E+00	4.64E-01	0.03
Glycine, serine and threonine metabolism	33	0.45	2	7.18E-02	1.14E+00	1.00E+00	4.64E-01	0.25
Nitrogen metabolism	6	0.08	1	7.87E-02	1.10E+00	1.00E+00	4.72E-01	0.00
Arginine and proline metabolism	38	0.51	2	9.18E-02	1.04E+00	1.00E+00	5.14E-01	0.09
Tryptophan metabolism	41	0.56	2	1.04E-01	9.81E-01	1.00E+00	5.38E-01	0.20
Tyrosine metabolism	42	0.57	2	1.09E-01	9.63E-01	1.00E+00	5.38E-01	0.02
Vitamin B6 metabolism	9	0.12	1	1.16E-01	9.36E-01	1.00E+00	5.40E-01	0.49
Nicotinate and nicotinamide metabolism	15	0.20	1	1.86E-01	7.31E-01	1.00E+00	7.88E-01	0.19
Histidine metabolism	16	0.22	1	1.97E-01	7.06E-01	1.00E+00	7.88E-01	0.00
Glycerolipid metabolism	16	0.22	1	1.97E-01	7.06E-01	1.00E+00	7.88E-01	0.04
Glycerophospholipid metabolism	36	0.49	1	3.91E-01	4.07E-01	1.00E+00	1.00E+00	0.08
Amino sugar and nucleotide sugar metabolism	37	0.50	1	4.00E-01	3.98E-01	1.00E+00	1.00E+00	0.00
Pyrimidine metabolism	39	0.53	1	4.16E-01	3.80E-01	1.00E+00	1.00E+00	0.05
Primary bile acid biosynthesis	46	0.62	1	4.71E-01	3.27E-01	1.00E+00	1.00E+00	0.01
Fatty acid biosynthesis	47	0.64	1	4.78E-01	3.20E-01	1.00E+00	1.00E+00	0.00
Purine metabolism	65	0.88	1	5.96E-01	2.25E-01	1.00E+00	1.00E+00	0.00

Table S5. Pathway analysis on down-regulated metabolites in DMSO vehicle treated conditioned media (CM_Vehicle) of LoVo compared to control media without cells (Control).

The Total is the total number of compounds in the pathway; the Hits is the actually matched number from the user uploaded data; the Raw p is the original p value calculated from the enrichment analysis; the Holm p is the p value adjusted by Holm-Bonferroni method; the FDR p is the p value adjusted using False Discovery Rate; the Impact is the pathway impact value calculated from pathway topology analysis.

	Total	Expected	Hits	Raw p	-log10(p)	Holm adjust	FDR	Impact
Aminoacyl-tRNA biosynthesis	48	0.81	12	1.29E-12	1.19E+01	1.08E-10	1.08E-10	0.00
Valine, leucine and isoleucine biosynthesis	8	0.13	3	2.22E-04	3.65E+00	1.84E-02	9.33E-03	0.00
Phenylalanine, tyrosine and tryptophan biosynthesis	4	0.07	2	1.59E-03	2.80E+00	1.30E-01	4.45E-02	1.00
Taurine and hypotaurine metabolism	8	0.13	2	7.12E-03	2.15E+00	5.77E-01	1.50E-01	0.71
Alanine, aspartate and glutamate metabolism	28	0.47	3	1.04E-02	1.98E+00	8.32E-01	1.57E-01	0.34
Phenylalanine metabolism	10	0.17	2	1.12E-02	1.95E+00	8.86E-01	1.57E-01	0.36
Glycine, serine and threonine metabolism	33	0.55	3	1.64E-02	1.79E+00	1.00E+00	1.97E-01	0.09
Biosynthesis of unsaturated fatty acids	36	0.60	3	2.07E-02	1.68E+00	1.00E+00	2.03E-01	0.00
Arginine biosynthesis	14	0.23	2	2.18E-02	1.66E+00	1.00E+00	2.03E-01	0.00
Nicotinate and nicotinamide metabolism	15	0.25	2	2.49E-02	1.60E+00	1.00E+00	2.09E-01	0.00
Histidine metabolism	16	0.27	2	2.81E-02	1.55E+00	1.00E+00	2.15E-01	0.22
beta-Alanine metabolism	21	0.35	2	4.67E-02	1.33E+00	1.00E+00	3.27E-01	0.00
Linoleic acid metabolism	5	0.08	1	8.12E-02	1.09E+00	1.00E+00	5.25E-01	1.00
D-Glutamine and D-glutamate metabolism	6	0.10	1	9.67E-02	1.01E+00	1.00E+00	5.41E-01	0.00
Nitrogen metabolism	6	0.10	1	9.67E-02	1.01E+00	1.00E+00	5.41E-01	0.00
Cysteine and methionine metabolism	33	0.55	2	1.04E-01	9.83E-01	1.00E+00	5.46E-01	0.13
Pyrimidine metabolism	39	0.65	2	1.37E-01	8.62E-01	1.00E+00	6.33E-01	0.01
Ubiquinone and other terpenoid-quinone biosynthesis	9	0.15	1	1.42E-01	8.49E-01	1.00E+00	6.33E-01	0.00
Valine, leucine and isoleucine degradation	40	0.67	2	1.43E-01	8.44E-01	1.00E+00	6.33E-01	0.00
Biotin metabolism	10	0.17	1	1.56E-01	8.07E-01	1.00E+00	6.55E-01	0.00
Pantothenate and CoA biosynthesis	19	0.32	1	2.76E-01	5.59E-01	1.00E+00	1.00E+00	0.00
Selenocompound metabolism	20	0.34	1	2.89E-01	5.40E-01	1.00E+00	1.00E+00	0.00
Purine metabolism	65	1.09	2	2.98E-01	5.25E-01	1.00E+00	1.00E+00	0.02
Lysine degradation	25	0.42	1	3.47E-01	4.60E-01	1.00E+00	1.00E+00	0.00
Glycolysis / Gluconeogenesis	26	0.44	1	3.58E-01	4.46E-01	1.00E+00	1.00E+00	0.00
Glyoxylate and dicarboxylate metabolism	32	0.54	1	4.21E-01	3.75E-01	1.00E+00	1.00E+00	0.00
Arginine and proline metabolism	38	0.64	1	4.78E-01	3.20E-01	1.00E+00	1.00E+00	0.01
Tryptophan metabolism	41	0.69	1	5.05E-01	2.97E-01	1.00E+00	1.00E+00	0.14
Tyrosine metabolism	42	0.70	1	5.13E-01	2.90E-01	1.00E+00	1.00E+00	0.14
Primary bile acid biosynthesis	46	0.77	1	5.46E-01	2.63E-01	1.00E+00	1.00E+00	0.01

Table S6. Pathway analysis on up-regulated metabolites in DMSO vehicle treated conditioned media (CM_Vehicle) of LoVo compared to control media without cells (Control).

The Total is the total number of compounds in the pathway; the Hits is the actually matched number from the user uploaded data; the Raw p is the original p value calculated from the enrichment analysis; the Holm p is the p value adjusted by Holm-Bonferroni method; the FDR p is the p value adjusted using False Discovery Rate; the Impact is the pathway impact value calculated from pathway topology analysis.

	Total	Expected	Hits	Raw p	-log10(p)	Holm adjust	FDR	Impact
Citrate cycle (TCA cycle)	20	0.32	5	9.45E-06	5.02E+00	7.94E-04	7.94E-04	0.23
Arginine biosynthesis	14	0.23	4	4.74E-05	4.32E+00	3.93E-03	1.54E-03	0.35
Alanine, aspartate and glutamate metabolism	28	0.45	5	5.49E-05	4.26E+00	4.50E-03	1.54E-03	0.33
Glyoxylate and dicarboxylate metabolism	32	0.52	5	1.08E-04	3.97E+00	8.72E-03	2.26E-03	0.13
Pyruvate metabolism	22	0.35	4	3.17E-04	3.50E+00	2.54E-02	5.33E-03	0.24
Butanoate metabolism	15	0.24	3	1.49E-03	2.83E+00	1.17E-01	2.08E-02	0.00
D-Glutamine and D-glutamate metabolism	6	0.10	2	3.60E-03	2.44E+00	2.81E-01	4.32E-02	0.50
Glutathione metabolism	28	0.45	3	9.31E-03	2.03E+00	7.17E-01	9.78E-02	0.11
Glycine, serine and threonine metabolism	33	0.53	3	1.47E-02	1.83E+00	1.00E+00	1.37E-01	0.25
Pantothenate and CoA biosynthesis	19	0.31	2	3.61E-02	1.44E+00	1.00E+00	3.03E-01	0.01
Aminoacyl-tRNA biosynthesis	48	0.77	3	3.97E-02	1.40E+00	1.00E+00	3.03E-01	0.00
Riboflavin metabolism	4	0.06	1	6.30E-02	1.20E+00	1.00E+00	4.14E-01	0.50
Glycolysis / Gluconeogenesis	26	0.42	2	6.41E-02	1.19E+00	1.00E+00	4.14E-01	0.10
Porphyrin and chlorophyll metabolism	30	0.48	2	8.25E-02	1.08E+00	1.00E+00	4.95E-01	0.00
Nitrogen metabolism	6	0.10	1	9.31E-02	1.03E+00	1.00E+00	5.11E-01	0.00
Cysteine and methionine metabolism	33	0.53	2	9.73E-02	1.01E+00	1.00E+00	5.11E-01	0.10
Thiamine metabolism	7	0.11	1	1.08E-01	9.67E-01	1.00E+00	5.33E-01	0.00
Taurine and hypotaurine metabolism	8	0.13	1	1.22E-01	9.13E-01	1.00E+00	5.46E-01	0.00
Arginine and proline metabolism	38	0.61	2	1.23E-01	9.09E-01	1.00E+00	5.46E-01	0.09
Vitamin B6 metabolism	9	0.15	1	1.36E-01	8.65E-01	1.00E+00	5.55E-01	0.49
Tryptophan metabolism	41	0.66	2	1.40E-01	8.54E-01	1.00E+00	5.55E-01	0.20
Tyrosine metabolism	42	0.68	2	1.45E-01	8.37E-01	1.00E+00	5.55E-01	0.02
Nicotinate and nicotinamide metabolism	15	0.24	1	2.17E-01	6.63E-01	1.00E+00	7.73E-01	0.19
Histidine metabolism	16	0.26	1	2.30E-01	6.38E-01	1.00E+00	7.73E-01	0.00
Glycerolipid metabolism	16	0.26	1	2.30E-01	6.38E-01	1.00E+00	7.73E-01	0.04
Glycerophospholipid metabolism	36	0.58	1	4.47E-01	3.50E-01	1.00E+00	1.00E+00	0.08
Pyrimidine metabolism	39	0.63	1	4.74E-01	3.24E-01	1.00E+00	1.00E+00	0.05
Primary bile acid biosynthesis	46	0.74	1	5.32E-01	2.74E-01	1.00E+00	1.00E+00	0.01

Table S7. Overlap of fold change of metabolite levels in DMSO vehicle treated conditioned media (CM_Vehicle) of HCT116 and LoVo compared to control media without cells (Control).

Metabolite	log2 (fold change, down-regulated)		Metabolite	log2 (fold change, up-regulated)	
	HCT116	LoVo		HCT116	LoVo
Hypoxanthine	-6.49	-6.48	Glycerol 3-phosphate	5.94	3.45
Niacin / nicotinate	-5.64	-6.73	Alpha-Ketoglutarate	3.95	4.10
Linoleic acid	-4.79	-2.89	Orotic acid	3.80	6.29
Cytidine	-4.69	-5.38	Pyruvate	3.25	2.86
Glutamine	-4.45	-4.78	Lactate	2.91	2.97
Serine	-2.28	-2.53	N-acetylaspartate	2.85	3.54
Taurine	-1.90	-2.09	Serotonin	1.59	1.99
Oleic acid	-1.74	-1.43	pyridoxal	1.26	1.93
Palmitoleic acid	-1.44	-1.91	acetyllysine	0.94	1.67
Tryptophan	-1.39	-2.05	Malate	0.61	0.72
Creatine	-1.39	-1.71	Fumarate	0.45	0.53
Leucine	-1.01	-0.68	glutamate	0.40	0.22
Lysine	-0.99	-0.85	Glycine	0.34	0.22
Methionine	-0.94	-1.29	L-Kynurenine	0.24	0.40
Threonine	-0.80	-0.98	Creatinine	0.19	0.24
Glucose	-0.69	-0.83			
Tyrosine	-0.66	-0.85			
L-Alanine	-0.50	-1.32			
L-Sarcosine	-0.50	-1.32			
Histidine	-0.50	-0.65			
Phenylalanine	-0.49	-0.67			
Cysteine sulfinic acid	-0.47	-0.76			
IsoLeucine	-0.46	-0.73			
carnitine	-0.45	-0.75			
Aspartate	-0.36	-0.72			

Table S8. Fold change of metabolite levels in 5-FU treated conditioned media (CM_5-FU) of HCT116 compared to DMSO vehicle treated conditioned media (CM_Vehicle).

Metabolite	log2 (fold change, down-regulated)	Metabolite	log2 (fold change, up-regulated)
Orotic acid	-1.84	Glutamine	4.02
Glycerol 3-phosphate	-1.02	Niacin/ Nicotinate	2.96
Lactate	-1.01	Hypoxanthine	2.24
Acetyllysine	-0.53	Octanoyl-carnitine	2.21
Asparagine	-0.51	Palmitoleic acid	1.74
Alpha-Ketoglutarate	-0.46	Serine	1.66
N-acetylaspartate	-0.25	Oleic acid	1.55
Fructose	-0.20	Taurine	1.55
Glutamate	-0.18	Tryptophan	1.18
Creatinine	-0.17	Lysine	0.88
		Creatine	0.84
		Ornithine	0.83
		Pyruvate	0.79
		Methionine	0.71
		Leucine	0.69
		Valine	0.64
		Cystine	0.57
		Threonine	0.56
		Tyrosine	0.50
		L-Alanine	0.44
		L-Sarcosine	0.44
		Cytidine	0.42
		Glucose	0.39
		Histidine	0.38
		Phenylalanine	0.37
		IsoLeucine	0.33
		Carnitine	0.24

Table S9. Fold change of metabolite levels in 5-FU treated conditioned media (CM_5-FU) of LoVo compared to DMSO vehicle treated conditioned media (CM_Vehicle).

Metabolite	log2 (fold change, down-regulated)	Metabolite	log2 (fold change, up-regulated)
Cysteine	-1.46	Docosahexaenoic acid	4.59
Butyric acid	-1.08	Glutamine	4.03
Acetyllysine	-0.93	Niacin/ Nicotinate	1.85
Lactate	-0.79	Oxoadipate	1.78
Serine	-0.77	Threonine	1.65
Folate	-0.67	Pyruvate	1.65
Cis-aconitate	-0.57	Hypoxanthine	1.51
Acetylcysteine	-0.45	Cytidine	1.49
L-Kynurenine	-0.35	Dodecanoic acid/Lauric acid	1.35
Citrulline	-0.34	L-Alanine	1.26
Carnosine	-0.25	L-Sarcosine	1.26
Fructose	-0.24	Creatine	1.09
Hexanoic acid	-0.24	Phenylalanine	1.05
Betaine	-0.22	Methionine	0.93
		Linoleic acid	0.78
		Lysine	0.77
		Oleic acid	0.76
		Serotonin	0.70
		Myristic acid	0.60
		Tryptophan	0.55
		Palmitoleic acid	0.50
		IsoLeucine	0.40
		Aspartate	0.32

Table S10. Pathway analysis on the overlap of metabolites in 5-FU treated conditioned media (CM_5-FU) of HCT116 and LoVo compared to DMSO vehicle treated conditioned media (CM_Vehicle).

The Total is the total number of compounds in the pathway; the Hits is the actually matched number from the user uploaded data; the Raw p is the original p value calculated from the enrichment analysis; the Holm p is the p value adjusted by Holm-Bonferroni method; the FDR p is the p value adjusted using False Discovery Rate; the Impact is the pathway impact value calculated from pathway topology analysis (maximum importance of each pathway is 1.0).

	Total	Expected	Hits	Raw p	-log10(p)	Holm adjust	FDR	Impact
Aminoacyl-tRNA biosynthesis	48	0.59	9	8.85E-10	9.05E+00	7.43E-08	7.43E-08	0.00
Glycine, serine and threonine metabolism	33	0.40	4	5.28E-04	3.28E+00	4.38E-02	2.22E-02	0.09
Phenylalanine, tyrosine and tryptophan biosynthesis	4	0.05	2	8.42E-04	3.07E+00	6.91E-02	2.36E-02	1.00
Valine, leucine and isoleucine biosynthesis	8	0.10	2	3.82E-03	2.42E+00	3.09E-01	7.09E-02	0.00
Alanine, aspartate and glutamate metabolism	28	0.34	3	4.22E-03	2.37E+00	3.37E-01	7.09E-02	0.11
Phenylalanine metabolism	10	0.12	2	6.04E-03	2.22E+00	4.78E-01	8.46E-02	0.36
Glyoxylate and dicarboxylate metabolism	32	0.39	2	5.68E-02	1.25E+00	1.00E+00	4.84E-01	0.00
Linoleic acid metabolism	5	0.06	1	5.99E-02	1.22E+00	1.00E+00	4.84E-01	1.00
Cysteine and methionine metabolism	33	0.40	2	6.00E-02	1.22E+00	1.00E+00	4.84E-01	0.10
Biosynthesis of unsaturated fatty acids	36	0.44	2	7.00E-02	1.15E+00	1.00E+00	4.84E-01	0.00
D-Glutamine and D-glutamate metabolism	6	0.07	1	7.14E-02	1.15E+00	1.00E+00	4.84E-01	0.00
Nitrogen metabolism	6	0.07	1	7.14E-02	1.15E+00	1.00E+00	4.84E-01	0.00
Arginine and proline metabolism	38	0.47	2	7.70E-02	1.11E+00	1.00E+00	4.84E-01	0.01
Pyrimidine metabolism	39	0.48	2	8.06E-02	1.09E+00	1.00E+00	4.84E-01	0.01
Tyrosine metabolism	42	0.51	2	9.17E-02	1.04E+00	1.00E+00	5.13E-01	0.14
Ubiquinone and other terpenoid-quinone biosynthesis	9	0.11	1	1.05E-01	9.77E-01	1.00E+00	5.53E-01	0.00
Biotin metabolism	10	0.12	1	1.16E-01	9.34E-01	1.00E+00	5.75E-01	0.00
Arginine biosynthesis	14	0.17	1	1.59E-01	7.98E-01	1.00E+00	7.43E-01	0.00
Nicotinate and nicotinamide metabolism	15	0.18	1	1.70E-01	7.71E-01	1.00E+00	7.50E-01	0.00
Purine metabolism	65	0.80	2	1.88E-01	7.26E-01	1.00E+00	7.89E-01	0.02
Selenocompound metabolism	20	0.25	1	2.20E-01	6.58E-01	1.00E+00	8.39E-01	0.00
Citrate cycle (TCA cycle)	20	0.25	1	2.20E-01	6.58E-01	1.00E+00	8.39E-01	0.05
Pyruvate metabolism	22	0.27	1	2.39E-01	6.21E-01	1.00E+00	8.73E-01	0.21
Lysine degradation	25	0.31	1	2.67E-01	5.73E-01	1.00E+00	9.28E-01	0.00
Glycolysis / Gluconeogenesis	26	0.32	1	2.76E-01	5.59E-01	1.00E+00	9.28E-01	0.10
Valine, leucine and isoleucine degradation	40	0.49	1	3.93E-01	4.05E-01	1.00E+00	1.00E+00	0.00
Tryptophan metabolism	41	0.50	1	4.01E-01	3.97E-01	1.00E+00	1.00E+00	0.14

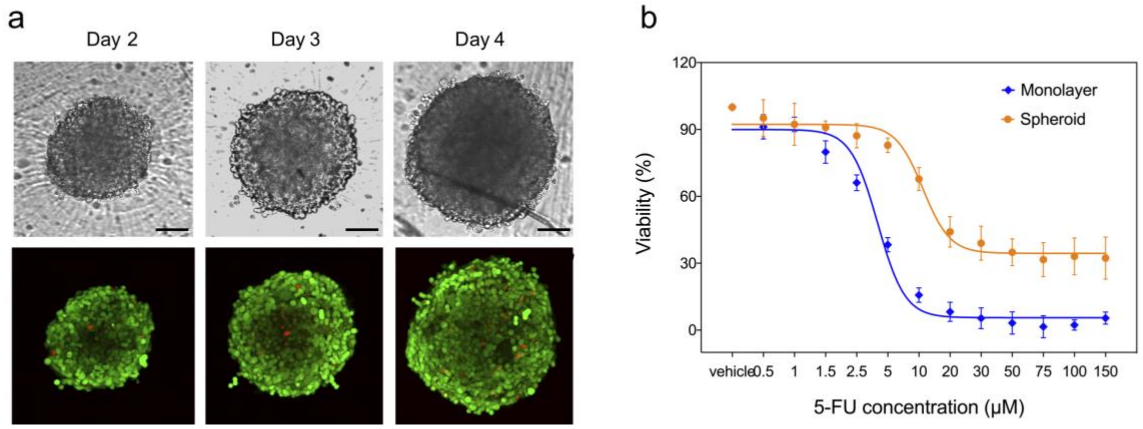

Figure S1 3D tumor spheroid formation of HCT116 and drug responses to 5-FU

(**a**) Representative live cell confocal fluorescence microscopy images showing HCT116 spheroid formation from day 2 to day 4 post seeding. Cell viability was assessed by live/dead staining using Calcein (green) and PI (red). Scale bars=100 μm. (**b**). Dose responses of HCT116 cells to 5-FU. Cells were treated with different concentrations of 5-FU and cell viability was measured using MTS assays. The squares, circles and triangles represent measured viability normalized to the vehicle control, while the solid lines represent dose response curves. The symbols represent the average and the error bars represent the standard deviation (n=3).

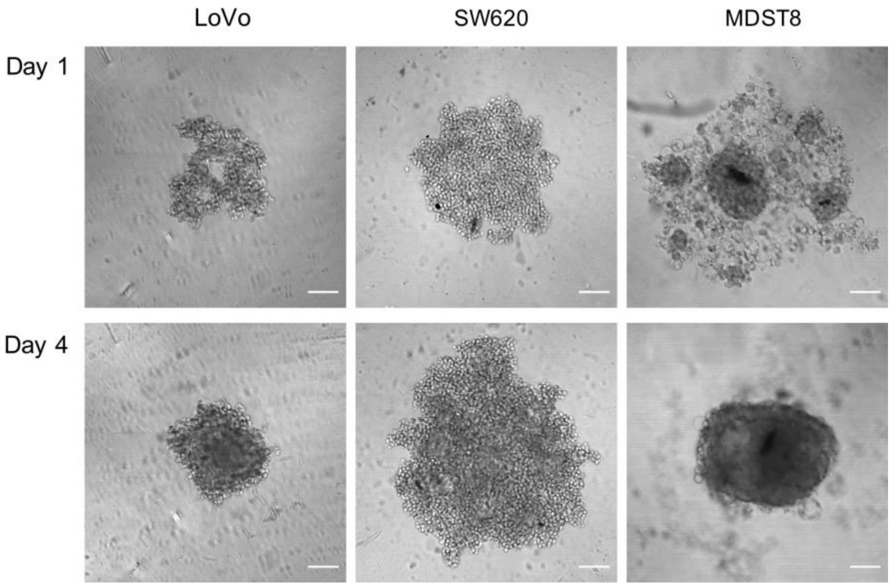

Figure S2 3D tumor spheroid formation of CMS cell lines

Representative bright field microscopy images showing LoVo, SW620 and MDST8 spheroid formation on day 1 and day 4 post seeding. Scale bars=100 μm.

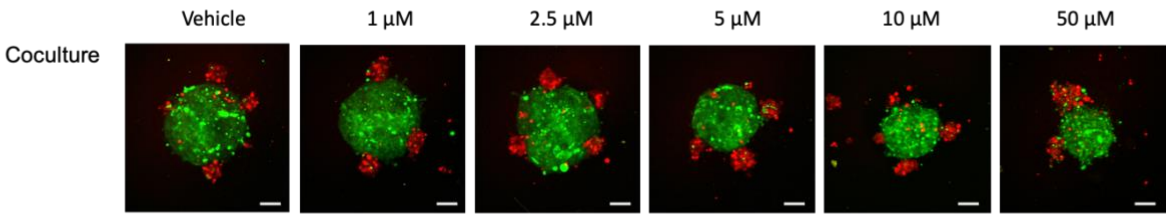

Figure S3 Cocultured spheroids of HCT116 and MDST8

Representative live cell confocal fluorescence microscopy images showing spheroid morphology after 3 days of 5-FU treatment. HCT116 cells were stained with cell tracker CMFDA (green) fluorescent probes and SW620 cells were stained with CMRA (red) fluorescent probes. Scale bars=100 µm.

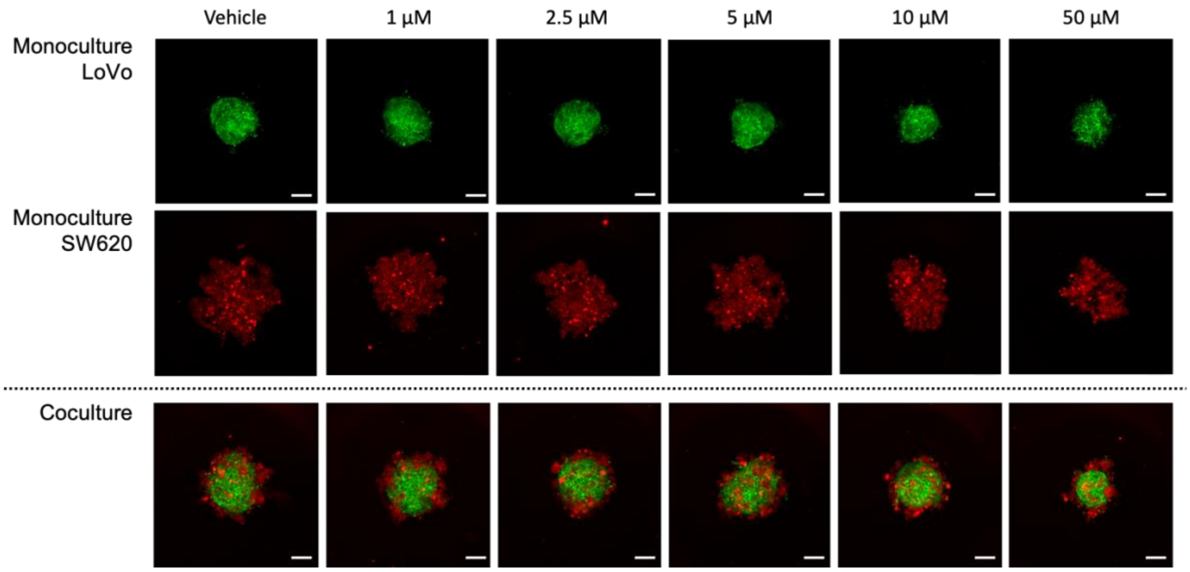

Figure S4 Monocultured and cocultured spheroids of LoVo and SW620

Representative live cell confocal fluorescence microscopy images showing spheroid morphology after 3 days of 5-FU treatment. LoVo cells expressed a GFP reporter (green) and SW620 cells were stained with cell tracker CMRA (red) fluorescent probes. Scale bars=100 μm.

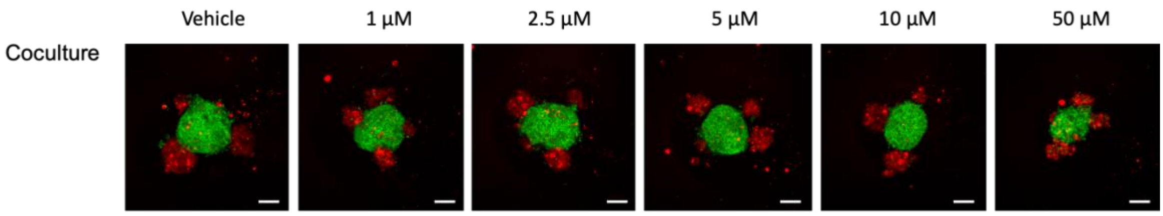

Figure S5 Cocultured spheroids of LoVo and MDST8

Representative live cell confocal fluorescence microscopy images showing spheroid formation morphology after 3 days of 5-FU treatment. LoVo cells expressed a GFP reporter (green) and MDST8 cells were stained with cell tracker CMRA (red) fluorescent probes. Scale bars=100 µm.

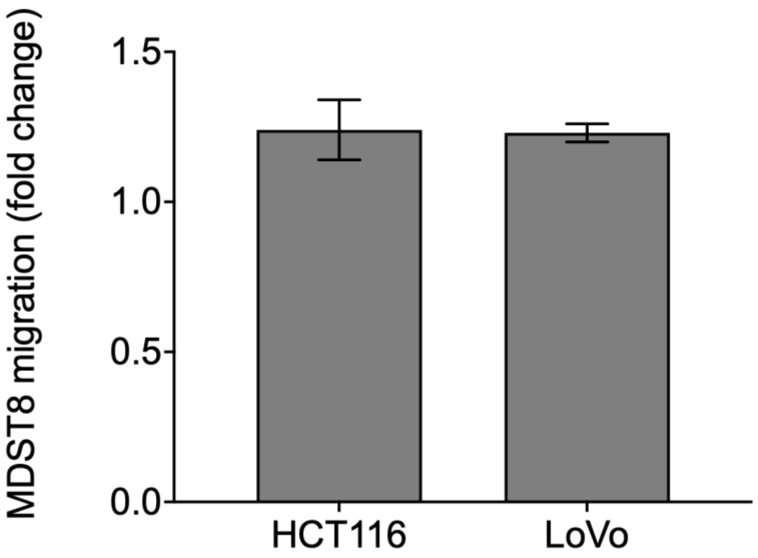

Figure S6 MDST8 cell migration through transwell membrane

Fold change of MDST8 migration through non-Matrigel coated transwell membrane when exposed to HCT116 or LoVo in the bottom wells. MDST8 exposed to only media without cells was taken as a control. Cells were treated with DMSO vehicle. The bars represent the average and the error bars represent the standard deviation (n=3). Statistical significance was calculated using a one-way ANOVA followed by Student's t-test. P-values of less than 0.05 and 0.01 were considered significant (*) and highly significant (**), respectively.

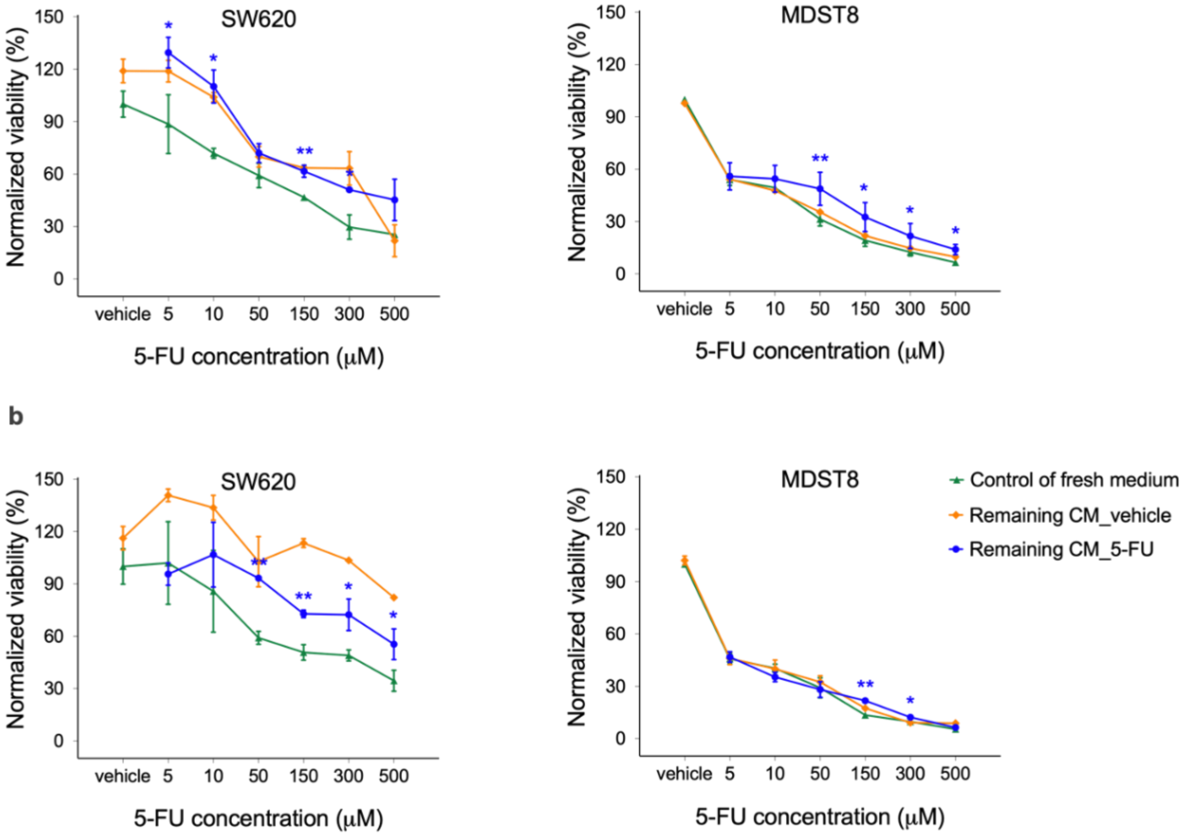

Figure S7 Drug resistance effect of remaining CMS1 conditioned media (CM)

CMS4 cells were treated with either remaining CM_vehicle or remaining CM_5-FU after the dialysis of metabolites, of (**a**) HCT116 or (**b**) LoVo CMS1 cells, and were exposed to different concentrations of 5-FU for 3 days. Cells treated with media only was taken as a control. Cell viability was measured using MTS assays. The squares, circles and triangles represent the average viability normalized to the control and the error bars represent the standard deviation (n=3). Statistical significance was calculated using a one-way ANOVA followed by Student's t-test. P-values of less than 0.05 and 0.01 were considered significant (*) and highly significant (**), respectively, when compared to the control.

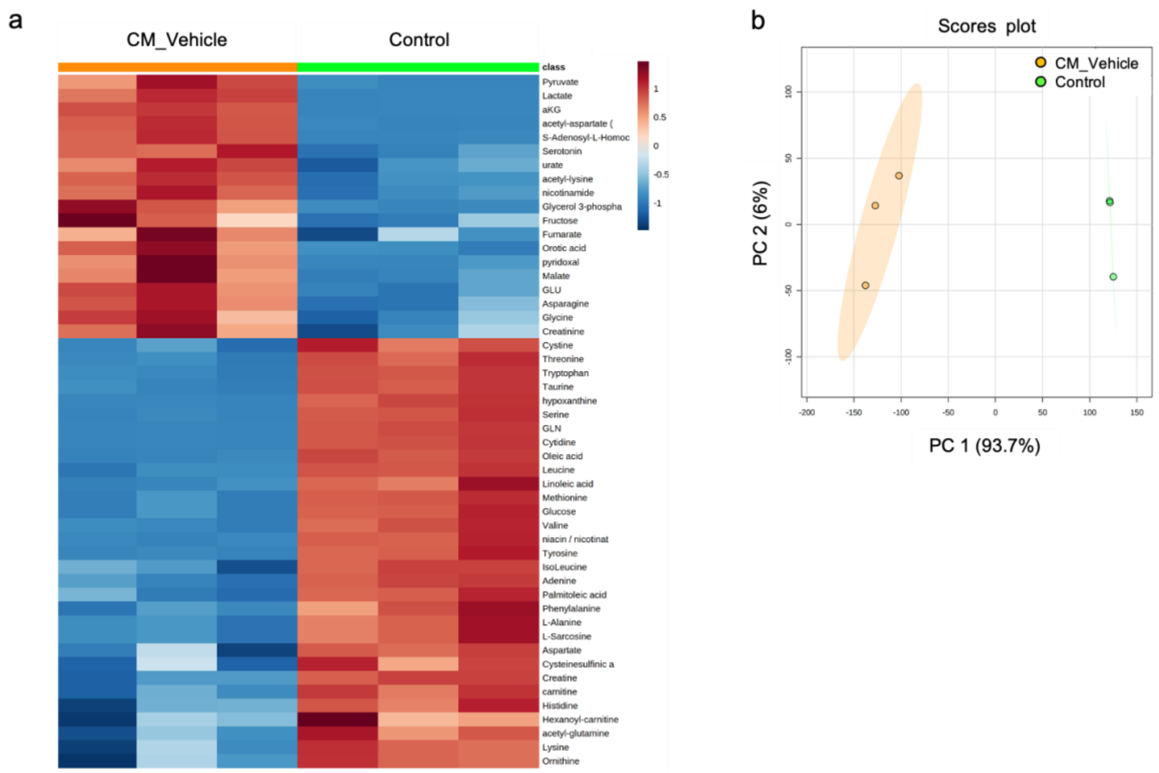

Figure S8 Metabolite analyses of HCT116 conditioned media

(**a**) Metabolomics profile of vehicle treated conditioned media (CM_Vehicle, orange) of HCT116 compared to control media without cells (Control, green), presented as a heatmap visualization and hierarchical clustering analysis. Rows are metabolites and columns are samples (n=3). The color key indicates the metabolite expression value (blue, lowest; red, highest). (**b**) Principal component analysis (PCA) plotting showing clusters of samples based on their similarity. Statistical significance was calculated using a one-way ANOVA followed by Student's t-test ($p<0.05$).

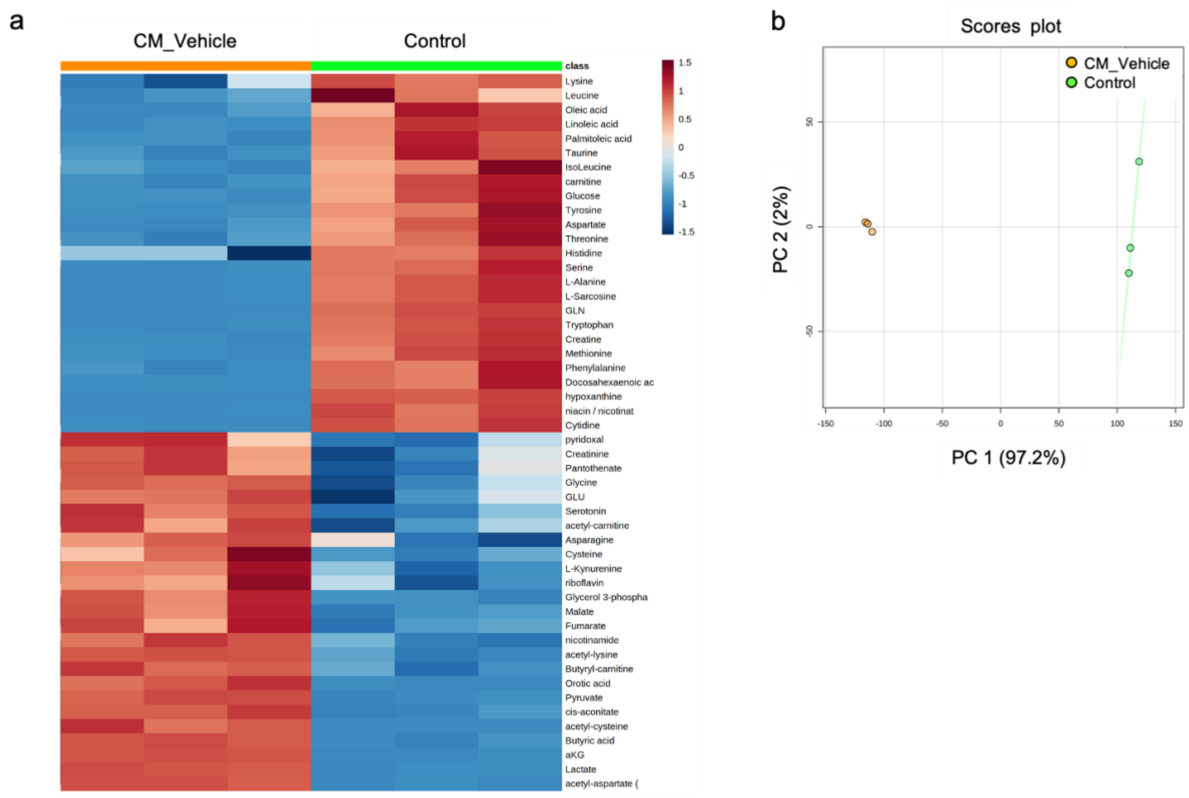

Figure S9 Metabolite analyses of DMSO vehicle treated conditioned media of LoVo

(**a**) Metabolomics profile of DMSO vehicle treated conditioned media (CM_Vehicle, orange) of LoVo compared to control media without cells (Control, green), presented as a heatmap visualization and hierarchical clustering analysis. Rows are metabolites and columns are samples (n=3). The color key indicates the metabolite expression value (blue, lowest; red, highest). (**b**) Principal component analysis (PCA) plotting showing clusters of samples based on their similarity. Statistical significance was calculated using a one-way ANOVA followed by Student's t-test (p<0.05).

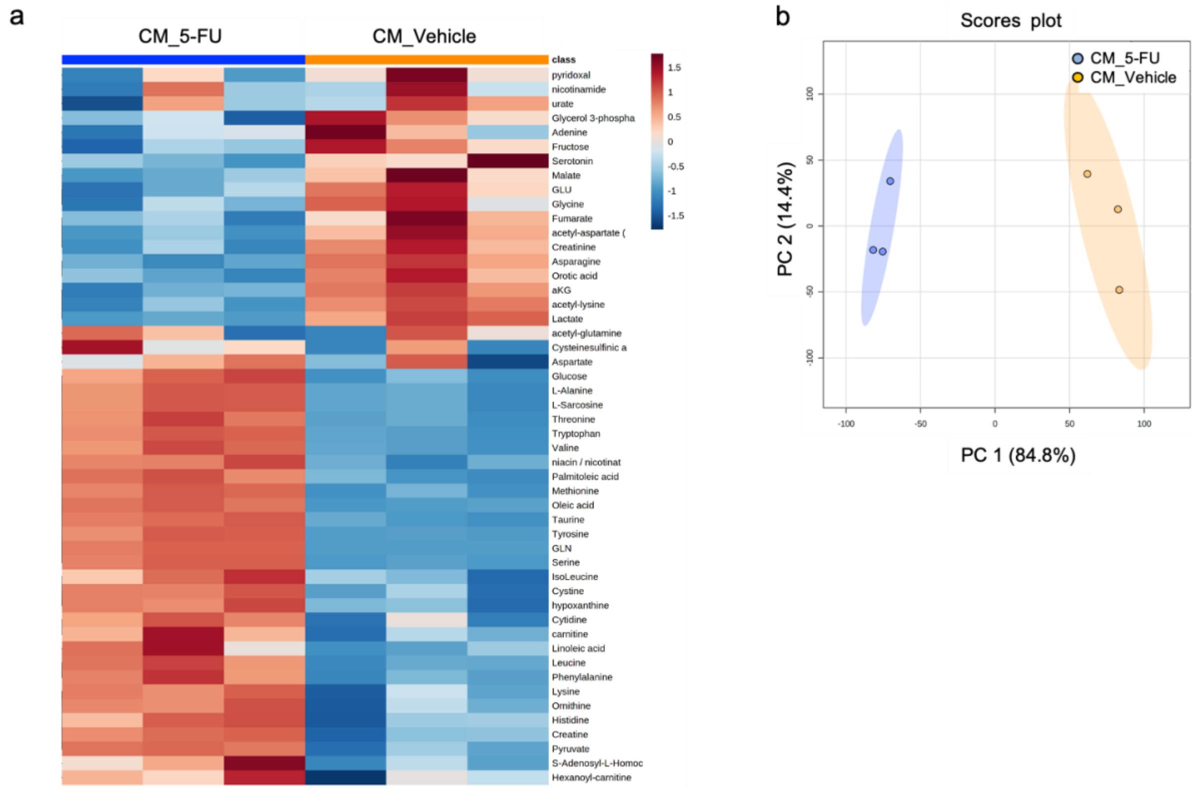

Figure S10 Metabolite analyses of 5-FU treated conditioned media of HCT116

(**a**) Metabolomics profile of 5-FU treated conditioned media (CM_5-FU, blue) of HCT116 compared to DMSO vehicle treated conditioned media (CM_Vehicle, orange), presented as a heatmap visualization and hierarchical clustering analysis. Rows are metabolites and columns are samples (n=3). The color key indicates the metabolite expression value (blue, lowest; red, highest). (**b**) Principal component analysis (PCA) plotting showing clusters of samples based on their similarity. Statistical significance was calculated using a one-way ANOVA followed by Student's t-test ($p<0.05$).

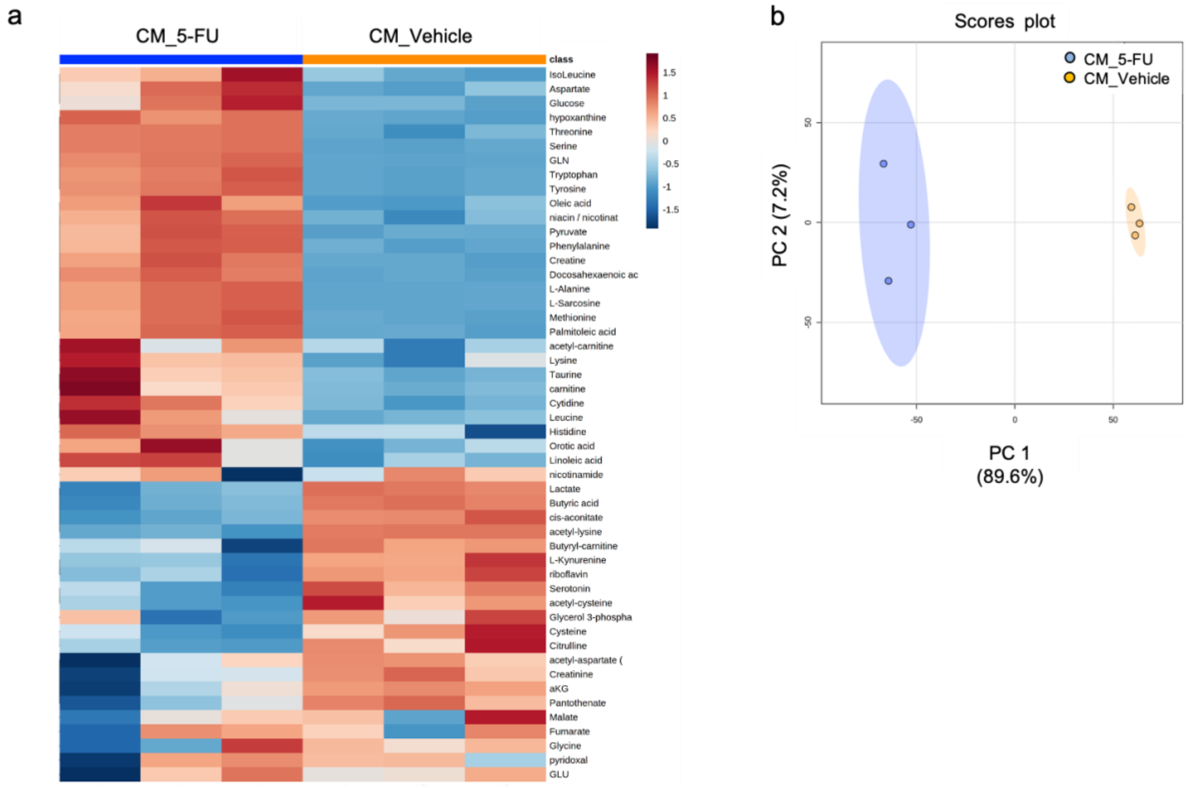

Figure S11 Metabolite analyses of 5-FU treated conditioned media of LoVo

(**a**) Metabolomics profile of 5-FU treated conditioned media (CM_5-FU, blue) of LoVo compared to DMSO vehicle treated conditioned media (CM_Vehicle, orange), presented as a heatmap visualization and hierarchical clustering analysis. Rows are metabolites and columns are samples from 3 independent experiments (n=3). The color key indicates the metabolite expression value (blue, lowest; red, highest). (**b**) Principal component analysis (PCA) plotting showing clusters of samples based on their similarity. Statistical significance was calculated using a one-way ANOVA followed by Student's t-test ($p<0.05$).

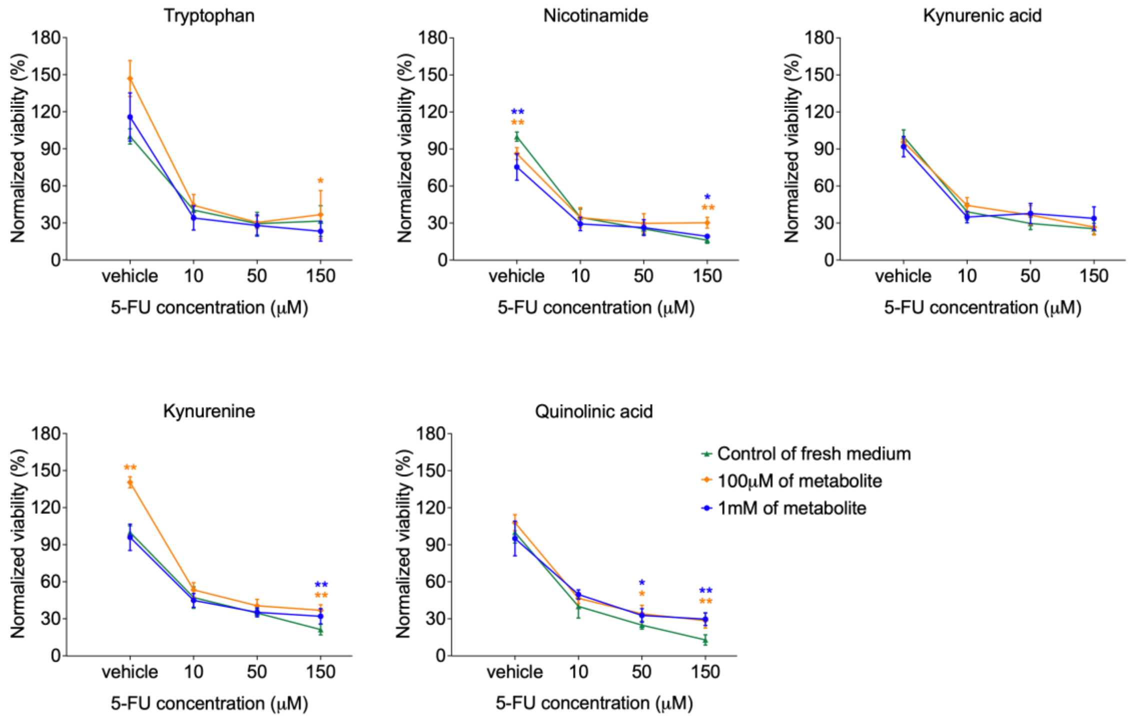

Figure S12 Drug resistance effect of metabolites in the kynurenine pathway on SW620

CMS4 cells were treated with either 100 μM or 1 mM of metabolite, and were exposed to different concentrations of 5-FU for 3 days. Cells treated with media only was taken as a control. Cell viability was measured using MTS assays. The squares, circles and triangles represent the average viability normalized to the control and the error bars represent the standard deviation (n=3). Statistical significance was calculated using a one-way ANOVA followed by Student's t-test. P-values of less than 0.05 and 0.01 were considered significant (*) and highly significant (**), respectively, when compared to the control.

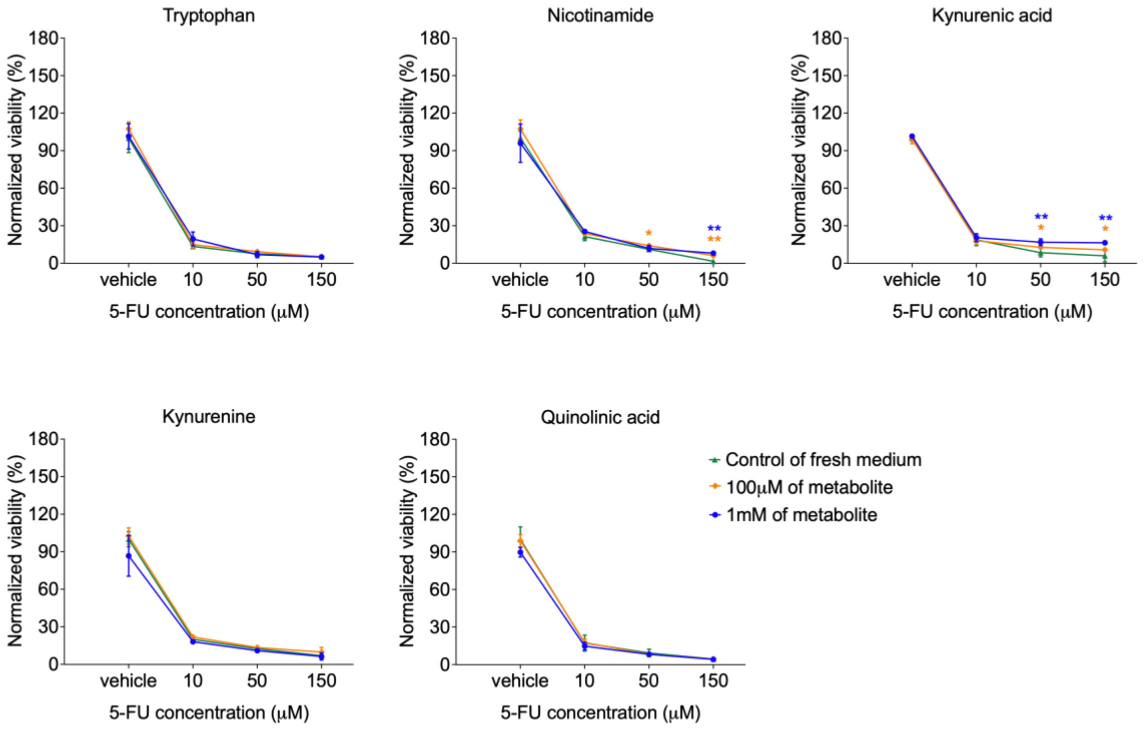

Figure S13 Drug resistance effect of metabolites in the kynurenine pathway on MDST8

CMS4 cells were treated with either 100 μM or 1 mM of metabolite, and were exposed to different concentrations of 5-FU for 3 days. Cells treated with media only was taken as a control. Cell viability was measured using MTS assays. The squares, circles and triangles represent the average viability normalized to the control and the error bars represent the standard deviation (n=3). Statistical significance was calculated using a one-way ANOVA followed by Student's t-test. P-values of less than 0.05 and 0.01 were considered significant (*) and highly significant (**), respectively, when compared to the control.
