# Supplementary material for: Intratumor heterogeneity and cell secretome promote chemotherapy resistance and progression of colorectal cancer
Source: Cell Death Dis. 2023 May 5;14(5):306. doi: 10.1038/s41419-023-05806-z (PMC10160076; doi:10.1038/s41419-023-05806-z)
Supplement: Supplementary file 2 — Supplemental table S2 [file 41419_2023_5806_MOESM2_ESM.pdf]

| Metabolite | CAS | KEGG |
|---|---|---|
| 3-hydroxybutyric acid | 300-85-6 | C01089 |
| 5-Hydroxylysine | | |
| Acetyl CoA | | C00024 |
| N-Acetyl-L-aspartic acid | 997-55-7 | |
| acetyl-carnitine | 3040-38-8 | C02571 |
| Acetylcholine | 51-84-3 | C01996 |
| Acetylcysteine | 616-91-1 | C06809 |
| N-Acetylglutamine | | |
| N6-Acetyl-L-lysine | | |
| Adenine | 73-24-5 | C00147 |
| Adenosine | 58-61-7 | C00212 |
| ADP | 58-64-0 | C00008 |
| alpha-Ketoglutaric acid | 328-50-7 | C00026 |
| Aminoadipate | 542-32-5 | C00956 |
| AMP | 61-19-8 | C00020 |
| Arachidonic acid | 506-32-1 | C00219 |
| Arginine | 74-79-3 | C00062 |
| Argininosuccinate | 2387-71-5 | C03406 |
| Asparagine | 70-47-3 | C00152 |
| Aspartate | 56-84-8 | C00049 |
| ATP | 56-65-5 | C00002 |
| beta Alanine | 107-95-9 | C00099 |
| Betaine | 107-43-7 | C00719 |
| Butyric acid | 107-92-6 | C00246 |
| Butyrylcarnitine | | C02862 |
| Caprylic acid | 124-07-2 | C06423 |
| Carnitine | 541-15-1 | C00318 |
| Carnosine | 305-84-0 | C00386 |
| CDP | 63-38-7 | C00112 |
| Choline | 62-49-7 | C00114 |
| cis-aconitate | 585-84-2 | C00417 |
| Citrate | 77-92-9 | C00158 |
| Citrulline | 372-75-8 | C00327 |
| CMP | 63-37-6 | C00055 |
| Coenzyme A | 85-61-0 | C00010 |
| Creatine | 57-00-1 | C00300 |
| Creatinine | 60-27-5 | C00791 |
| CTP | 65-47-4 | C00063 |
| Cystathionine | 56-88-2 | C02291 |
| Cystine | 56-89-3 | C00491 |
| Cytidine | 65-46-3 | C00475 |
| Decanoic acid | 172,2646 | C01571 |
| Decanoylcarnitine | 1492-27-9 | |
| Dihydroxyacetone phosphate | 57-04-5 | C00111 |
| Docosahexaenoic acid | 6217-54-5 | C06429 |
| dodecanoic acid | 143-07-7 | C02679 |
| Dodecanoylcarnitine | 25518-54-1 | |
| Eicosapentaenoic acid | 10417-94-4 | |
| ethanolamine phosphate | 1071-23-4 | C00346 |
| FAD | 146-14-5 | C00016 |

| | | |
|---|---|---|
| Folate | 59-30-3 | C00504 |
| fructose 1,6-diphosphate | 488-69-7 | C00354 |
| Fumarate | 110-17-8 | C00122 |
| GDP | 146-91-8 | C00035 |
| Glutamine | 56-85-9 | C00064 |
| Glutamate | 6893-26-1 | C00217 |
| Glucose | 50-99-7 | C00031 |
| Glucose-6-phosphate | 56-73-5 | C00092 |
| D-Glyceraldehyde 3-phosphate | 142-10-9 | C00661 |
| Glycerol 3-phosphate | 57-03-4 | C00093 |
| Glycine | 56-40-6 | C00037 |
| GMP | 85-32-5 | C00144 |
| GSH | 70-18-8 | C00051 |
| GSSG | 27025-41-8 | C00127 |
| GTP | 86-01-1 | C00044 |
| Guanine | 73-40-5 | C00242 |
| Guanosine | 118-00-3 | C00387 |
| Hexanoic acid | 142-62-1 | C01585 |
| Hexanoyl-L-carnitine | 6418-78-6 | |
| Histidine | 71-00-1 | C00135 |
| Homocysteine | 6027-13-0 | C00155 |
| Hydroxy-L-proline | 51-35-4 | C01157 |
| Hypoxanthine | 68-94-0 | C00262 |
| IMP | 131-99-7 | C00130 |
| Inosine | 58-63-9 | C00294 |
| IsoLeucine | 73-32-5 | C00407 |
| Lactate | 79-33-4 | C00186 |
| L-Alanine | 56-41-7 | C00041 |
| L-alpha-Amino-N-butyric acid | 1492-24-6 | C02356 |
| L-beta-Aminoisobutyric acid | 4249-19-8 | C03284 |
| Leucine | 61-90-5 | C00123 |
| Linoleic acid | 60-33-3 | C01595 |
| Linolenic acid | 463-40-1 | C06427 |
| L-Sarcosine | 107-97-1 | C00213 |
| Lysine | 56-87-1 | C00047 |
| Malate | 97-67-6 | C00149 |
| Methionine | 63-68-3 | C00073 |
| N(6)-Methyllysine | 1188-07-4 | C02728 |
| 1-Methylhistidine | 368-16-1 | C01152 |
| Myristic acid | 544-63-8 | C06424 |
| Myristoylcarnitine | 25597-07-3 | |
| NAD+ | 53-84-9 | C00003 |
| NADH | 58-68-4 | C00004 |
| NADP+ | 53-59-8 | C00006 |
| NADPH | 53-57-6 | C00005 |
| Nicotinate | 59-67-6 | C00253 |
| Nicotinamide | 98-92-0 | C00153 |
| Nicotinamide N-oxide | 1986-81-8 | |
| L-Octanoylcarnitine | 25243-95-2 | C02838 |
| Oleic acid | 112-80-1 | C00712 |
| O-Phosphoethanolamine | 1071-23-4 | C00346 |
| O-Phospho-L-serine | 17885-08-4 | C01005 |

| | | |
|---|---|---|
| Ornithine | 70-26-8 | C00077 |
| Oxoadipate | 3184-35-8 | C00322 |
| Oxypurinol | 2465-59-0 | C07599 |
| Palmitic acid | 57-10-3 | C00249 |
| Palmitoleic acid | 373-49-9 | C08362 |
| Palmitoylcarnitine | 2364-67-2 | C02990 |
| Pantothenate | 79-83-4 | C00864 |
| 2-phosphoenolpyruvate | 138-08-9 | C00074 |
| Phenol red | 143-74-8 | |
| Phenylalanine | 63-91-2 | C00079 |
| Phosphocreatine | 67-07-2 | C02305 |
| picolinate | | |
| Proline | 147-85-3 | C00148 |
| Propionyl-carnitine | 17298-37-2 | C03017 |
| pyridoxal | | |
| Pyruvate | 127-17-3 | C00022 |
| Riboflavin | 83-88-5 | C00255 |
| Ribose | 50-69-1 | C00121 |
| Ribose 1-phosphate | 14075-00-4 | C00620 |
| S-Adenosyl-L-Homocysteine | 979-92-0 | C00021 |
| S-Adenosyl-L-methionine | 29908-03-0 | C00019 |
| D-Sedoheptulose 7-phosphate | 2646-35-7 | C05382 |
| Serine | 56-45-1 | C00065 |
| Stearic acid | 57-11-4 | C01530 |
| Succinic acid | 110-15-6 | C00042 |
| S-Cysteinosuccinic acid | | |
| S-succinylglutathione | | |
| Taurine | 107-35-7 | C00245 |
| Thiamine | | |
| Threonine | 72-19-5 | C00188 |
| Thymidine | | |
| Tryptophan | 73-22-3 | C00078 |
| Tyrosine | 60-18-4 | C00082 |
| UDP | 58-98-0 | C00015 |
| UDP-GlcNAc | 528-04-1 | C00043 |
| UMP | 58-97-9 | C00105 |
| Urate | 69-93-2 | C00366 |
| Uridine | 58-96-8 | C00299 |
| Uridine diphosphate glucose | 133-89-1 | C00029 |
| UTP | 63-39-8 | C00075 |
| Valine | 72-18-4 | C00183 |
| Xanthine | 69-89-6 | C00385 |
| Xanthosine | | |
| Xanthylic acid | | |
